# Development and Calibration of a Phenomenological Material Model for Steel-Fiber-Reinforced High-Performance Concrete Based on Unit Cell Calculations

**DOI:** 10.3390/ma17102247

**Published:** 2024-05-10

**Authors:** Mangesh Pise, Dominik Brands, Jörg Schröder

**Affiliations:** Institute of Mechanics, Faculty of Engineering, Department Civil Engineering, University of Duisburg-Essen, Universitätsstr. 12, 45141 Essen, Germany; dominik.brands@uni-due.de (D.B.); j.schroeder@uni-due.de (J.S.)

**Keywords:** ellipsoidal representative volume element (RVE), fiber-reinforced high-performance concrete, phase-field modeling, fracture of concrete, macroscopic model

## Abstract

A phenomenological material model has been developed to facilitate the efficient numerical analysis of fiber-reinforced high-performance concrete (HPC). The formulation integrates an elasto-plastic phase-field model for simulating fractures within the HPC matrix, along with a superimposed one-dimensional elasto-plasticity model that represents the behavior of the embedded fibers. The Drucker–Prager plasticity and one-dimensional von-Mises plasticity formulations are incorporated to describe the nonlinear material behavior of both the HPC matrix and the fibers, respectively. Specific steps are undertaken during the development and calibration of the phenomenological material model. In the initial step, an experimental and numerical analysis of the pullout test of steel fibers embedded in an HPC matrix is conducted. This process is used to calibrate the micro-mechanical model based on the elasto-plastic phase-field formulation for fracture. In the subsequent step, virtual experiments based on an ellipsoidal unit cell, also with the resolution of fibers (used as a representative volume element, RVE), are simulated to analyze the impact of fiber–matrix interactions and their physical properties on the effective material behavior of fiber-reinforced HPC. In the final step, macroscopic boundary value problems (BVPs) based on a cuboid are simulated on a single scale using the developed phenomenological material model. The resulting macroscopic stress–strain characteristics obtained from both types of simulations, namely simulations of virtual experiments and macroscopic BVPs, are compared. This comparison is utilized for the calibration of material parameters to obtain a regularized solution and to assess the effectiveness of the presented phenomenological material model.

## 1. Introduction

In recent years, reinforced high-performance concrete (HPC) has emerged as a specialized construction material known for its exceptional mechanical properties, including its high strength, durability, toughness, and ductility, see [1]. These qualities make it a preferred material for a wide range of structural applications, including, but not limited to, highways, tunnels, high-rise buildings, bridges, drainage systems, and nuclear facilities, see [2,3]. Modern HPCs have captured the attention of researchers and engineers in the civil industry due to their diverse compositions, which distinguish them from conventional concrete types and endow them with exceptional properties. However, it is important to note that the real strength of reinforced HPC comes from its reinforcement. Reinforcement plays a crucial role in distributing stress evenly throughout the material, which disables further crack propagation and significantly enhances its capacity to withstand bending, see [4]. High-strength steel fibers are commonly used as reinforcements to increase the tensile strength and ductility of HPC, see [5]. In the failure process of fiber-reinforced HPC, the stresses within the concrete are transferred from the matrix to the fibers, which greatly contributes to the concrete’s energy absorption capacity, as discussed in [6,7]. The effectiveness of fibers in transferring applied stresses primarily depends on the properties of the fiber–matrix interface, see [8]. Consequently, the efficiency of fiber reinforcement is principally determined by the interaction between the fibers and the surrounding concrete matrix, cf. [9,10]. Anothercrucial aspect contributing to the exceptional properties of fiber-reinforced HPC is its heterogeneous microstructure, cf. [11]. This microstructure is characterized by a dense matrix, refined pores, and well-distributed reinforcement materials. The complex interactions between fibers and the HPC matrix at the microscale dominate the macroscopic behavior of fiber-reinforced HPC during failure, as discussed in [12]. As the effects of these interactions are not yet fully understood, more comprehensive research is required to achieve a thorough understanding of the behavior of HPC at the microstructural level and the significant influence of embedded fibers on the material’s overall performance, particularly for fiber-reinforced HPC subjected to cyclic loading. Both experimental and numerical analyses are essential for gaining a comprehensive understanding of and optimizing the material behavior of fiber-reinforced HPC, especially in cases where these materials are subjected to cyclic loading. In pursuit of this goal, the German Research Foundation Priority Programme 2020 (DFG SPP 2020) has been founded, with the authors of this paper collaborating on a collective project called ”Effects of Steel-fibers on the Degradation of High-Performance Concrete subjected to Fatigue Loading-Testing and Modeling” that focuses on the experimental and numerical investigation of fatigue failure in HPC. This initiative aims to advance our understanding and optimization of fiber-reinforced HPCs, with the ultimate goal of enabling their more effective utilization in practical applications.

The numerical investigation of concrete failure has always been a fascinating and critical area of research. Researchers are continually working on advancing continuum damage models to better understand and predict the complex mechanical responses of concrete during failure, e.g., see [13,14,15,16,17,18,19,20]. These models are designed to accurately capture the nonlinear behavior of concrete materials under various loading conditions. The recently developed phase-field approach for fracture has demonstrated its capability to approximate the complex failure mechanisms and the propagation of cracks. In the context of a phase-field model for fracture, the Γ-convergent approximation is a fundamental mathematical concept that plays a pivotal role in approximating the energy functional. This approach, as introduced in [21,22], is utilized by researchers such as in [23] to regularize the variational approach for modeling fracture in brittle materials as proposed in [24]. The phase-field approach for fracture enables the modeling of crack propagation and fracture by representing cracks as continuous, diffuse phase boundaries rather than sharp interfaces. The effectiveness of this formulation is evident in recently developed fracture models employing the phase-field approach for various applications, e.g., for simulation of fracture in brittle materials approximating the crack as an individual phase, see [23,25,26,27,28,29,30,31,32,33,34,35]; for the prediction of complex crack propagation in the case of dynamic fracture, see [36,37,38,39]; and for elasto-plastic phase-field formulations to capture the failure behavior of pseudo-ductile materials, see [40,41,42,43,44,45,46,47,48,49,50].

The phase-field approach for modeling fracture has gained significant prominence because it eliminates the need for tracking crack paths explicitly. This characteristic makes phase-field models effective in predicting complex crack propagation in heterogeneous materials like rocks, soils, and concretes, cf. [51,52,53,54,55,56]. However, the phase-field model demands a finely discretized domain as a fundamental requirement. This leads to limitations when applying the direct homogenization methods like the FE^2^ method, cf. [57], in conjunction with the phase-field model. Hence, it becomes imperative to conduct further research and investigations that rely on homogenization techniques, particularly when numerically simulating fiber-reinforced HPC containing a substantial number of fibers using the phase-field model.

Therefore, the main aim of this paper is to develop and calibrate a phenomenological material model, which represents the fibers within the HPC matrix numerically. To simplify the complexities associated with the combination of the phase-field approach and the direct homogenization methods, we adopt a step-by-step approach. The steps that are followed include:

- Numerical calibration of a micro-mechanical model using single-fiber pullout tests;
- Analysis of the effective macroscopic behavior of fiber-reinforced HPC through virtual experiments based on unit cell calculations using a micro-mechanical model;
- Calibration and validation of the phenomenological material model by comparing the macroscopic responses obtained from macroscopic BVPs and virtual experiments.

**Figure 1:**
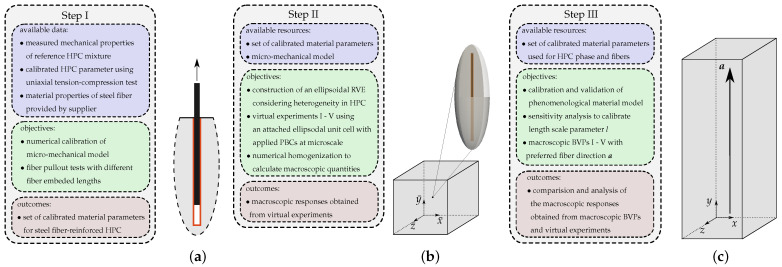
Graphical representation of the step-by-step approach: representative boundary value problems (BVPs) (**a**) fiber pullout test in step I, (**b**) virtual experiments in step II and (**c**) macroscopic BVPs in step III.

The steps described above are shown graphically in Figure 1 to explain the procedure. The figure illustrates how the data and findings obtained from the preceding steps are utilized to achieve the objectives of the current steps. Additionally, representative boundary value problems (BVPs) associated with these steps are depicted in Figure 1a–c, respectively.

To perform efficient numerical analyses of fiber-reinforced HPC, it is crucial to have a comprehensive understanding of the interactions between fibers and the matrix, as well as the microstructure and properties of HPC. The single-fiber pullout test for steel fibers embedded in HPC is a widely accepted test in the research community, with the primary goal of evaluating the bond strength between individual steel fibers and the HPC matrix. Numerous experimental and numerical studies on the single-fiber pullout test have been conducted and documented in the literature, e.g., see [5,8,58,59,60,61,62,63,64,65]. For this reason, the analysis involves both experimental and numerical investigations of the pullout test with a steel fiber embedded in the HPC matrix. This entire process is utilized to calibrate the micro-mechanical model based on the elasto-plastic phase-field formulation for fracture. Comprehensive details can be found in references [66,67,68]. Therein, the primary goal was to develop a numerical model capable of accurately predicting the load–displacement characteristics observed in fiber pullout tests conducted in experiments.

In the next step, the macro-mechanical model is utilized to analyze the macroscopic behavior of the ellipsoidal RVEs consisting of pure HPC and reinforced HPC under various loading conditions. For this purpose, virtual experiments based on an individual ellipsoidal RVE are simulated. To achieve this objective, an ellipsoidal RVE is constructed which characterizes the behavior of steel fiber-reinforced HPC in the preferred fiber direction. In these simulations, the macro-mechanical model and the material parameters calibrated in the previous stage are employed. The homogenized macroscopic quantities are computed by taking the volume average of corresponding microscopic quantities and plotted in the macroscopic stress–strain diagram.

In the final step, the formulation of the phenomenological material model is developed. Therein, the combination of an elasto-plastic phase-field model to simulate fracture in the HPC matrix and a superimposed one-dimensional elasto-plasticity model characterizing the behavior of embedded fibers in the preferred fiber direction is used. To check the prediction capabilities of the phenomenological material model, macroscopic BVPs applying similar loading conditions to those of the corresponding virtual experiments are simulated. Finally, the resulting macroscopic stress–strain characteristics obtained from simulations of virtual experiments based on RVEs of pure and reinforced HPC and macroscopic BVPs using cuboids of pure and reinforced HPC are compared.

In this paper, the constitutive framework of the micro-mechanical model and the results of numerical simulations of pullout tests of single steel fibers embedded within the HPC matrix are documented in Section 2. The kinematics and concept of the numerical homogenization procedure used to compute the macroscopic quantities and the details of the construction of an ellipsoidal RVE are described in Section 3. Therein, the results obtained from simulating virtual experiments using the constructed ellipsoidal RVEs are thoroughly documented and discussed. In Section 4, the formulation of the phenomenological material model is documented and the efficiency of the developed model is discussed the simulations results of macroscopic BVPs. Lastly, the outcomes of the presented work are concluded in Section 5.

The presented numerical models are implemented in the framework of the Finite Element Method using the finite element analysis program FEAP (version 8.2) (A Finite Element Analysis Program by R.L. Taylor, UC, Berkeley), see [69].

## 2. Numerical Calibration of the Micro-Mechanical Model Using Fiber Pullout Tests

In this section, the formulation of the small strain elasto-plastic phase-field model for fracture in HPCs is documented, see our recent publications [67,68,70]. The model is calibrated to the experimental results for a pullout test of a single steel fiber embedded in HPC.

### 2.1. Constitutive Framework of a Small-Strain Elasto-Plastic Phase-Field Model for Fractures

In the context of a small strain, the displacement field u(x,t) and the phase-field parameter $q\in \left[0,1\right]$ are considered, for details, see [25]. It depicts the unbroken state at q=0 and the fully broken state at q=1 of the material. In the phase-field approach of fracture, a length-scale parameter *l* controls the regularization of the crack surface energy $\Gamma_{l}\left(q\right)$, i.e.,

(1) $$\Gamma_{l}\left(q\right)=\int_{B}\gamma \left(q,\nabla q\right)\;dv\;\mathrm{with}\;\gamma \left(q,\nabla q\right)=\frac{1}{2l}\;\;q^{2}+\frac{l}{2}{\;||\nabla q||}^{2}\;,$$

where ∇q is the gradient of the phase-field parameter *q* and γ(q,∇q) is called the crack surface density function per unit volume of the solid. The free energy function ψ can be constructed as,

(2) $$\psi \left(\varepsilon ,\varepsilon^{p},\alpha ,q,\nabla q\right)=\;\psi^{\mathrm{ep}}\left(\varepsilon^{e},\alpha ,q\right)+\psi^{c}-g\left(q,m\right)\;\psi^{c}+2\;\frac{\psi^{c}}{\zeta }\;l\;\gamma \left(q,\nabla q\right)\;,$$

where α is the equivalent plastic strain. The specific critical fracture energy $\psi^{c}>0$ serves as the threshold for crack evolution. The parameter ζ controls the stress softening in the post critical region, see [43]. A parameter *m* used in the degradation function $g\left(q,m\right)={\left(1-q\right)}^{m}$ controls the speed of fracture evolution, cf. [44,67,68,71]. The total strain tensor ε is defined using the symmetric displacement gradient $\nabla_{s}u$ as,

(3) $$\varepsilon \left(u\right)=\nabla_{s}u=\frac{1}{2}\left(\nabla u+\nabla^{T}u\right),$$

which is used along with plastic strain tensor $\varepsilon^{p}$ to calculate elastic strain tensor $\varepsilon^{e}$, i.e.,

(4) $$\varepsilon^{e}:=\varepsilon -\varepsilon^{p}.$$

An elastic energy $\psi^{e}$ and a plastic energy $\psi^{p}$ are parts of the elastic–plastic energy function $\psi^{\mathrm{ep}}$, i.e.,

(5) $$\psi^{\mathrm{ep}}=\psi^{e}\left(\varepsilon^{e}\right)+\psi^{p}\left(\alpha \right)\;.$$

An additive form of the considered elastic energy function $\psi^{e}$ can be formulated as,

(6) $$\psi^{e}\left(\varepsilon^{e},q\right)=g\left(q,m\right)\psi_{0}^{e+}\left(\varepsilon^{e}\right)+\psi_{0}^{e-}\left(\varepsilon^{e}\right)\;,$$

and using a positive part $\psi_{0}^{e+}\left(\varepsilon^{e}\right)$ and a negative part $\psi_{0}^{e-}\left(\varepsilon^{e}\right)$ of the reference energy function, i.e., $\psi_{0}^{e}\left(\varepsilon^{e}\right)=\psi_{0}^{e+}\left(\varepsilon^{e}\right)+\psi_{0}^{e-}\left(\varepsilon^{e}\right)$, as proposed in [72], respectively, this means that

(7) $$\psi_{0}^{e+}\left(\varepsilon^{e}\right)=\kappa \;{\langle \mathrm{tr}\left[\varepsilon^{e}\right]\rangle }_{+}^{2}/2+\mu \;||\mathrm{dev}\varepsilon^{e}{\left|\right|}^{2}\;\mathrm{and}\;\psi_{0}^{e-}\left(\varepsilon^{e}\right)=\kappa \;{\langle \mathrm{tr}\left[\varepsilon^{e}\right]\rangle }_{-}^{2}/2\;.$$

Therein, μ represents the shear modulus and κ is the bulk modulus. Macaulay’s notation is used to describe the function ${\langle •\rangle }_{\pm }=1/2\;\left(\;•\pm |•|\;\right)$. The plastic energy $\psi^{p}$ can be expressed using the reference plastic energy function $\psi_{0}^{p}\left(\alpha \right)$ which depends on the equivalent plastic strain α and the hardening parameter *h*, as

(8) $$\psi^{p}\left(\alpha \right)=g\left(q,m\right)\;\psi_{0}^{p}\left(\alpha \right),\;\mathrm{where}\;\psi_{0}^{p}\left(\alpha \right)=y_{0}\;\alpha +\frac{1}{2}h\;\alpha^{2}\;.$$

The energy density function ψ is restructured as, cf. [43],

(9) $$\psi \left(\varepsilon ,\varepsilon^{p},\alpha ,q,\nabla q\right)={\left(1-q\right)}^{m}\left[\psi_{0}^{e+}+\psi_{0}^{p}-\psi^{c}\right]+\psi_{0}^{e-}+\psi^{c}+2\;\frac{\psi^{c}}{\zeta }\;l\;\left[\frac{1}{2l}\;q^{2}+\frac{l}{2}{\;||\nabla q||}^{2}\right].$$

The equation of the stress tensor is

(10) $$\sigma :=\partial_{\varepsilon^{e}}\psi ={\left(1-q\right)}^{m}\underset{\sigma_{0}^{+}}{\underset{︸}{\left[\kappa {\langle \mathrm{tr}\;\varepsilon^{e}\rangle }_{+}I+2\mu \;\mathrm{dev}\varepsilon^{e}\right]}}+\underset{\sigma_{0}^{-}}{\underset{︸}{\left[\kappa {\langle \mathrm{tr}\;\varepsilon^{e}\rangle }_{-}I\right]}}\;,$$

where the symbol I denotes the second-order identity tensor. The positive $\sigma_{0}^{+}$ and the negative $\sigma_{0}^{-}$ stress tensor are parts of the effective stress tensor $\sigma_{0}=\sigma_{0}^{+}+\sigma_{0}^{-}$. The governing equation for the phase-field parameter is computed as

(11) $$q-l^{2}\;\mathrm{Div}\left[\nabla q\right]-\left(1-q\right)H=0\;.$$

Therein, to ensure the upper and lower bounds of the range of the phase-field parameter q∈[0,1], the parameter m=2 is set. The irreversibility of the evolution of cracks is ensured by considering the local history field H and the maximum value of a dimensionless crack driving state function $H_{0}$, cf. [73], i.e.,

(12) $$H:=\max_{\tilde{t}\in \left[0,t\right]}\;H_{0}\left(x,\tilde{t}\right)\geq 0\;\mathrm{where}\;H_{0}=\zeta \langle \frac{\psi_{0}^{e+}\left(\varepsilon^{e}\right)}{\psi^{c}}+\frac{\psi_{0}^{p}\left(\alpha \right)}{\psi^{c}}-1\rangle \;.$$

For loading–unloading processes, the update of the local history field at the time $t_{n+1}$ follows

(13) $$\begin{array}{c} H=\left\{\begin{array}{c} H_{0,n+1}\;\;\;\mathrm{for}\;\;H_{0,n+1}>H_{0,n}\;,\\ H_{0,n}\;\;\;\;\mathrm{otherwise}\;. \end{array}\right. \end{array}$$

The governing equation of the phase-field parameter *q*, see Equation (11), is driven by the maximum value of a dimensionless crack driving state function H, see Equation (12). The damage evolves if the total values of the positive part of the elastic energy $\psi_{0}^{e+}$ and the reference plastic energy $\psi_{0}^{p}$ overcome the critical value $\psi^{c}$, see Equation (12). The distinct behavior of concrete under increasing loads, i.e., the earlier failure of concrete in tension rather than in compression, can be captured by using two different critical fracture energies. To achieve this, distinct parameters for the critical fracture energy in tension, denoted as $\psi_{t}^{c}$, and in compression, denoted as $\psi_{c}^{c}$, are considered. These parameters are distinguished from each other based on the sign of the first invariant of the stress tensor using the condition:

(14) $$\begin{array}{c} \psi^{c}=\left\{\begin{array}{c} \psi_{t}^{c},\;\mathrm{for}\;\;\mathrm{tr}\;\varepsilon \geq 0\;,\\ \psi_{c}^{c},\;\mathrm{otherwise}\;. \end{array}\right. \end{array}$$

The presented numerical model incorporates the associative Drucker–Prager yield criterion, which is capable of predicting the distinct behavior of concrete in tensile and in compressive loading, cf. [74]. It reads

(15) $$\phi \left(\sigma_{0},\kappa_{p}\right)=\frac{1}{\sqrt{2}}\;||\mathrm{dev}\sigma_{0}\left|\right|+\beta_{p}\;\mathrm{tr}\;\sigma_{0}-\left(y_{0}+h\;\alpha \right)\;.$$

Note that the plastic response is independent of the evolution of the damage yield criterion, see Equation (15), which depends on the effective stress tensor $\sigma_{0}$, see Equation (10). This criterion provides flexibility to use the von Mises yield criterion by eliminating the hydrostatic stress component, i.e., $\beta_{p}=0$, see [74]. The von Mises yield criterion is

(16) $$\phi \left(\sigma_{0},\kappa_{p}\right)=\frac{1}{\sqrt{2}}\;||\mathrm{dev}\sigma_{0}\left|\right|-\left(y_{0}+h\;\alpha \right)\;.$$

Here, the values of the yield stress $y_{0}$ and hardening parameter *h* should be calibrated accordingly to achieve the well-known von Mises yield surface, cf. [75]. Thus, different yield criteria can be easily used for the prediction of the elasto-plastic behavior of steel fibers and a concrete matrix. The weak formulation of the balance of linear momentum using the stress tensor, see Equation (10), i.e.,

(17) $$\delta G_{u}\left(u,\delta u\right)=\int_{B}\delta \varepsilon :\sigma \;dv-\int_{\partial B_{t}}\;\delta u\cdot t\;da=0\;,$$

with the virtual strains $\delta \varepsilon =\frac{1}{2}\left(\nabla \delta u+\nabla^{T}\delta u\right)$, and of the governing equation for the phase-field parameter, see Equation (11), i.e.,

(18) $$\delta G_{q}\left(q,\delta q\right)=\int_{B}q\;\delta q\;dv+\int_{B}l^{2}\;\nabla q\cdot \nabla \delta q\;dv-\int_{B}\left(1-q\right)\;H\;\delta q\;dv=0\;,$$

are obtained using the standard Galerkin procedure. Therein, the boundary conditions σ·n=t on $\partial B_{t}$, the part of ∂B with prescribed traction boundary conditions, and ∇q·n=0 on the surface ∂B of the domain B are considered. The symbols n and t denote the normal vector and traction vector, respectively. These weak forms are solved using the framework of the Finite Element Method, see, e.g., [76]. The approximation of all fields (u,q) is achieved using the trilinear ansatz function, i.e., with eight-node hexahedrons. The domain is discretized in such a manner that the element size $h^{e}$ within the area of interest is less than half of the value of the length-scale parameters *l*. The time step $\Delta t=1\times 10^{-4}$ is selected according to a preliminary study which leads to the converged solution in time. In this context, the integration algorithm for the Drucker–Prager plasticity as well as the consistent elasto-plastic tangent moduli, as detailed in [70], has been implemented. The numerical solution is obtained using incrementally decoupled updates of the weak form of the field equations, i.e., Equations (17) and (18), as described in [25,77], and a staggered solution scheme explained in [40,78]. The supplementary data for the FE analysis of fiber pullout tests and virtual experiments are provided in [79,80], respectively.

### 2.2. Numerical Simulations of Pullout Tests of a Steel Fiber Embedded in HPC

**Figure 2:**
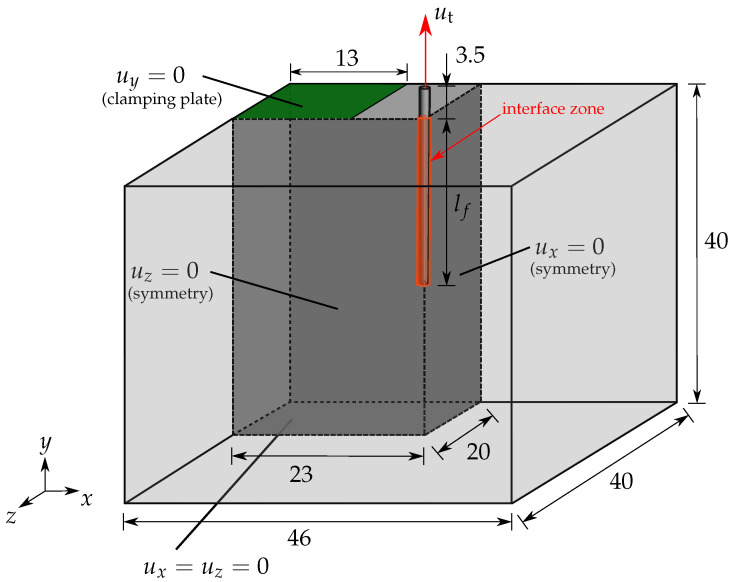
Boundary value problem for steel-fiber pullout tests based on the experimental assembly, adopted from [68]. All dimensions are in mm.

In this section, the procedure used for calibration and validation of a macro-mechanical model by simulating single-fiber pullout tests is documented. The geometry of the fiber pullout test setup and the boundary conditions adopted from the experimental assembly, see [66,68], are shown in Figure 2. Due to axial symmetry around the *x*- and *z*-axes, only a quarter of the full assembly, see the dark gray boundaries in Figure 2, is used for the numerical simulations. The displacement in the direction normal to the cutting planes is fixed. The bottom face is fixed in the *x*- and *z*-directions. The vertical displacements of region where the clamping plates are placed, see the green region in Figure 2, are fixed. A displacement boundary condition $u_{t}$ in the *y*-direction at the top of fiber is applied, see the red arrow in Figure 2. The sum of the reaction forces of the constrained nodes at the top surface of the fiber is computed and reported to plot load–displacement diagrams.

Steel fibers 3D55/60, provided by the supplier Bekaert GmbH, with a diameter of approximately 1 mm are used, see [67]. The thickness of the interface zone around the fiber, highlighted in orange in Figure 2, is taken as 0.5 mm. The embedded length of the fiber, i.e., the length of the interface zone, is denoted by $l_{f}$. The mechanical properties, i.e., Young’s modulus *E*, Poisson ratio μ and tensile strength $f_{t}$, of the steel fibers are provided by the supplier, see Table 1. The experimentally determined mechanical properties of HPC, i.e., Young’s modulus *E*, Poisson ratio μ, tensile strength $f_{t}$ and compressive strength $f_{c}$, are taken from [66], see Table 1. The von Mises yield criterion, see Equation (16), and the Drucker–Prager yield criterion, see Equation (15), are used for the nonlinear behavior of steel and HPC materials, respectively. The calculation of material parameters for steel and HPC related to plasticity, e.g., the initial yield stress $y_{0}$ and the Drucker–Prager parameter $\beta_{p}$, was conducted easily using the tensile strength $f_{t}$ and compressive strength $f_{c}$. The critical fracture energies in tension $\psi_{t}^{c}$ and in compression $\psi_{c}^{c}$ are taken from our works [68,81]. Therein, uniaxial cyclic tension and compression tests are simulated to calibrated these parameters and to determine the behavior of HPC in the softening region; for more details, see [68,70]. The specific critical fracture energy is assumed to not vary during tension and compression for the steel fibers and the interface zone, see Table 1, taken from [68]. The mechanical properties for fiber–matrix interface, i.e., Young’s modulus *E*, Poissons ratio ν and the initial yield stress $y_{0}$, are set to the same values for the HPC material. The von Mises yield criterion, see Equation (16), is considered for the elastic–plastic behavior of the interface material. A steel-fiber pullout test with an embedded length of $l_{f}\;=\;20$ mm is simulated and the results are compared with the experimental data to calibrate the rest of the material parameters for the interface.

**Figure 3:**
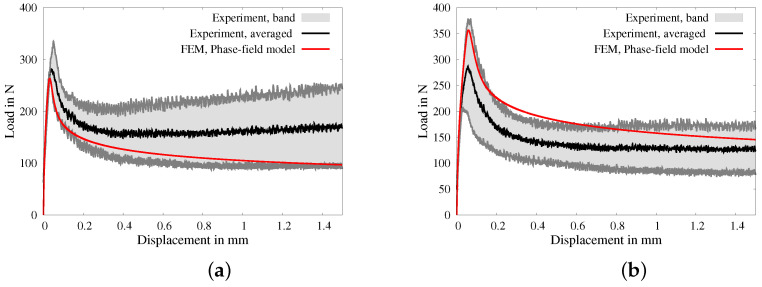
Comparison of load–displacement diagrams of simulations with the experimental averaged curves and scatter bands of experimental data, taken from [66], for an embedded length (**a**) $l_{f}=20\;\mathrm{mm}$ and (**b**) $l_{f}=30\;\mathrm{mm}$.

For the validation of the numerical model, a steel-fiber pullout test with an embedded length of $l_{f}=30\;\mathrm{mm}$ is simulated using the same set of parameters. The simulations of the fiber pullout test for an embedded length of fiber $l_{f}=20\;\mathrm{mm}$ and $l_{f}=30\;\mathrm{mm}$ are discretized with 29,889 and 54,366 elements, respectively. The resulting load–displacement diagrams for simulations with an embedded length of $l_{f}=20\mathrm{mm}$ and $l_{f}=30\;\mathrm{mm}$ are compared with experimental data in Figure 3a and Figure 3b, respectively. Therein, the experimental data are depicted through the averaged curves and their corresponding scatter bands, taken from [66]. The results for simulations with an embedded length of $l_{f}=20\;\mathrm{mm}$ and $l_{f}=30\;\mathrm{mm}$ fit into the respective experimental band, see Figure 3.

**Figure 4:**
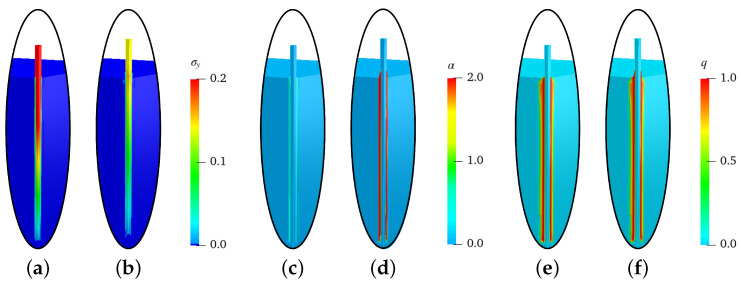
Simulation results of steel-fiber pullout tests for $l_{f}=20\;\mathrm{mm}$: stresses $\sigma_{y}$ (in GPa), equivalent plastic strains α and phase-field parameter *q* in (**a**,**c**,**e**) at a displacement of $u_{t}=0.08$ mm and (**b**,**d**,**f**) at a displacement of $u_{t}=0.8$ mm, respectively.

**Figure 5:**
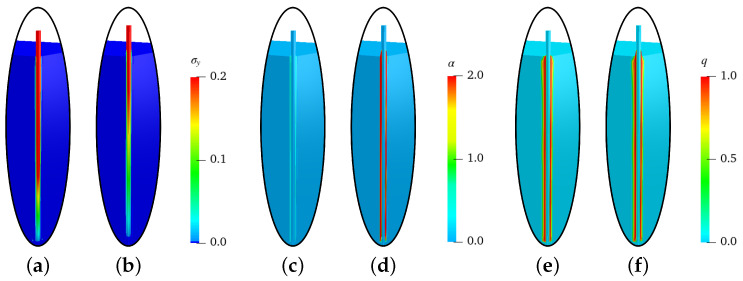
Simulation results of steel-fiber pullout test for $l_{f}=30\;\mathrm{mm}$: stresses $\sigma_{y}$ (in GPa), equivalent plastic strains α and phase-field parameter *q* in (**a**,**c**,**e**) at a displacement of $u_{t}=0.08$ mm and (**b**,**d**,**f**) at a displacement of $u_{t}=0.8$ mm, respectively.

In Figure 4 and Figure 5, the distributions of stresses in the *y*-direction $\sigma_{y}$ (in GPa), equivalent plastic strains α and the phase-field parameter *q* at the displacements of $u_{t}=0.08$ mm and $u_{t}=0.8$ mm for the simulations of steel-fiber pullout tests with an embedded length of $l_{f}=20\;\mathrm{mm}$ and $l_{f}=30\;\mathrm{mm}$ are shown, respectively. For the both solutions, the stresses in the fiber at $u_{t}=0.08$ mm are higher than those at $u_{t}=0.8$ mm, compare Figure 4a,b and Figure 5a,b. This is because the fiber stress reduces after debonding of the interface zone, i.e., after evolution of damage in the interface zone. The evolution of equivalent plastic strains α from the initial stage, i.e., at $u_{t}=0.08$ mm, to the later stage, i.e., at $u_{t}=0.8$ mm, can be observed in Figure 4c,d and Figure 5c,d. The damage initiates in the initial stage, i.e., at $u_{t}=0.08$, due to the shear band in the complete interface zone, then it evolves in the later stages, e.g., at $u_{t}=0.08$, depending on the evolution of the equivalent plastic strains α, see Figure 4e,f and Figure 5e,f. This shows the capability of the presented numerical model to predict the evolution of damage due to plasticity. For details on the different stages of fiber pullout tests, refer to [68].

## 3. Virtual Experiments Based on Ellipsoidal RVEs

This section details the construction of an ellipsoidal unit cell, taking into account the fiber reinforcement of the concrete material. It also outlines the numerical framework employed to analyze the failure behavior of both pure and reinforced HPC. This approach involves conducting the simulations of the virtual experiments based on ellipsoidal RVEs.

### 3.1. Kinematics and Concept of Numerical Homogenization 

**Figure 6:**
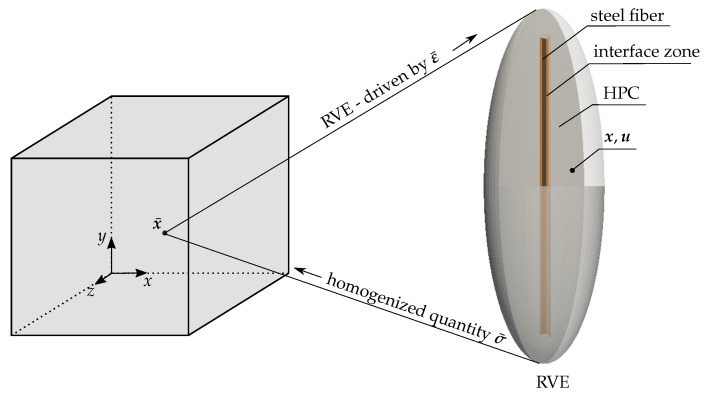
Macroscopic boundary value problem—an attached RVE at a macroscopic material point $\bar{x}$ driven by macroscopic strains $\bar{\varepsilon }$, adopted from [81].

To distinguish between the quantities at macro- and micro-scales, the macroscopic quantities are denoted by an overline $\left(\bar{•}\right)$. An ellipsoidal RVE containing the fiber and the HPC matrix is considered. This RVE is attached at each material point $\bar{x}$ at the macroscale. The boundary conditions at the microscale are derived using the Hill–Mandel condition, cf. [82,83]. The basic idea is to drive RVEs at the microscale by applying macroscopic strains $\bar{\varepsilon }$ and compute the microscopic field quantities, i.e., the strain ε and stress σ. Here, an elasto-plastic phase-field model using the Drucker–Prager plasticity formulation, illustrated in Section 2, is taken into account for computations at the microscale. The macroscopic quantities, e.g., stress $\bar{\sigma }$, are computed from the volume averages of their microscopic counterparts. The concept of the homogenization approach is illustrated in Figure 6.

The body $\bar{B}\in R^{3}$ in the Euclidean three-dimensional space $R^{3}$ is considered on the macroscale, which is parameterized by the position vector of a material point $\bar{x}$. The macroscopic symmetric linear strain tensor $\bar{\varepsilon }\left(\bar{x}\right)$ is given by the symmetric displacement gradient $\nabla_{s}\bar{u}$ of the macroscopic displacement $\bar{u}\left(\bar{x}\right)$, defined as

(19) $$\bar{\varepsilon }\left(\bar{x}\right)=\nabla_{s}\bar{u}\left(\bar{x}\right)=\frac{1}{2}\left(\nabla_{\bar{x}}\bar{u}\left(\bar{x}\right)+\nabla_{\bar{x}}^{T}\bar{u}\left(\bar{x}\right)\right)\;,$$

where $\nabla_{\bar{x}}$ denotes the gradient operator with respect to $\bar{x}$. The local form of the balance of linear momentum on the macroscale, neglecting the body forces $\bar{b}$ and acceleration terms $\ddot{\bar{x}}$, is

(20) $${\mathrm{Div}}_{\bar{x}}\;\bar{\sigma }=0\;.$$

The macroscopic boundary conditions are considered as $\bar{u}={\bar{u}}_{\bar{b}}$ and $\bar{t}=\bar{\sigma }\cdot \bar{n}$ on $\partial \bar{B},$ where $\bar{t}$ and $\bar{n}$ are traction vectors and the outward unit normal on the boundary $\partial \bar{B},$ respectively. ${\mathrm{Div}}_{\bar{x}}$ denotes the divergence operator with respect to $\bar{x}$. Analogously, on the microscale, the body of interest $B\in R^{3}$ is parameterized by the position vector of a material point x. The microscopic strain tensor ε(x) is given by the symmetric microscopic displacement gradient $\nabla_{s}u$, see Equation (3). On the macroscale, a constitutive model is not presumed. Instead of this, to get the constitutive response on the macroscale, an RVE is attached at each macroscopic point $\bar{x}$. The direct homogenization procedure is applied to obtain the macroscopic Cauchy stress tensor $\bar{\sigma }$ and strain tensor $\bar{\varepsilon }$ from their microscopic counterparts, cf. [57]. Therein, the suitable surface integrals over the boundary of the ∂B with volume *V* are considered. Neglecting the singular surface and holes in the RVE, the macroscopic field quantities can be computed by averaging the microscopic fields over the volume *V* of the RVE, i.e.,

(21) $$\bar{\varepsilon }:=\frac{1}{V}\int_{\partial B}\left[u⊗n\right]\;da=\frac{1}{V}\int_{B}\varepsilon \;dv\;\mathrm{and}\;\bar{\sigma }:=\frac{1}{V}\int_{\partial B}\left[t⊗x\right]\;da=\frac{1}{V}\int_{B}\sigma \;dv\;,$$

where t and n are the traction vector and outward unit normal on the boundary ∂B, respectively. Additive decomposition of the microscopic strain tensor consists of a constant $\bar{\varepsilon }$ and a fluctuation part $\tilde{\varepsilon }$. Based on the ansatz $u:=\bar{\varepsilon }\cdot x+\tilde{w}$, where $\tilde{w}$ denotes the fluctuations over the RVE, we obtain

(22) $$\varepsilon =\bar{\varepsilon }+\tilde{\varepsilon }\;\mathrm{with}\;\tilde{\varepsilon }=\nabla_{s}\tilde{w}\;\mathrm{and}\;\frac{1}{V}\int_{B}\tilde{\varepsilon }\;dv=\frac{1}{V}\int_{\partial B}\left[\tilde{w}⊗n\right]\;da=0\;,$$

**Figure 7:**
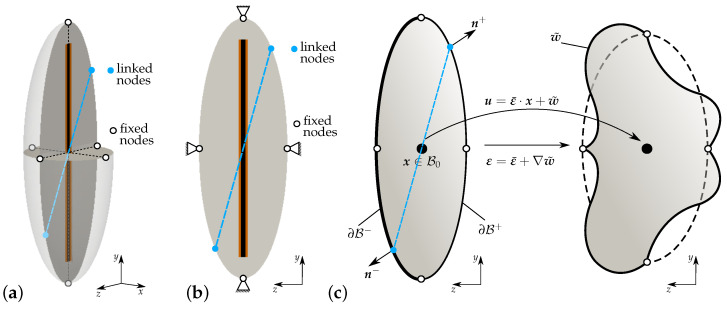
Applied periodic boundary conditions on the ellipsoidal RVE: (**a**) cross-sectional view, (**b**) cross-sectional plane, fixed nodes (black dots) on surface of an RVE and linked nodes (blue dots) on the boundary of a particular plane in an RVE, (**c**) illustration of the periodic boundary conditions applied on an ellipsoidal RVE for the simulations.

Appropriate boundary conditions of the boundary value problem at the microscale are derived from the macro-homogeneity condition, also known as the Hill–Mandel condition, cf. [82,83]. Possible boundary conditions are

(23) $$t=\sigma \cdot n\;\forall \;x\in \partial B\;\mathrm{or}\;\dot{u}=\dot{\bar{\varepsilon }}\cdot x\;\forall \;x\in \partial B$$

which denote the mechanical Neumann and Dirichlet boundary conditions on the RVE, respectively. Periodic boundary conditions (PBCs) satisfying the Hill–Mandel condition are

(24) $${\tilde{w}}^{+}\left(x^{+}\right)={\tilde{w}}^{-}\left(x^{-}\right),\;t^{+}\left(x^{+}\right)=-t^{-}\left(x^{-}\right)\;\mathrm{and}\;n^{+}=-n^{-}\;,$$

where equal fluctuation of deformation, opposing traction vectors and opposing normal vectors at associated points $x^{\pm }\in \partial B^{\pm }$ are denoted by the symbols $t^{\pm }$, ${\tilde{w}}^{\pm }$ and $n^{\pm }$, respectively. Figure 7 describes the applied periodic boundary conditions. In Figure 7a, the fixed nodes at the highest and lowest point along the *y*-axis and the nodes on the *x*-axis and *z*-axis at the boundary of the middle plane are shown using blue dots. In Figure 7b, for the application of periodic boundary conditions, the ellipsoidal RVE is discretized with exact point symmetry on the lateral surface. A more general concept for applying periodic boundary conditions can be found in [84]; generalized boundary conditions on RVEs are discussed in [85].

### 3.2. Ellipsoidal RVE for Fiber-Reinforced HPC

**Figure 8:**
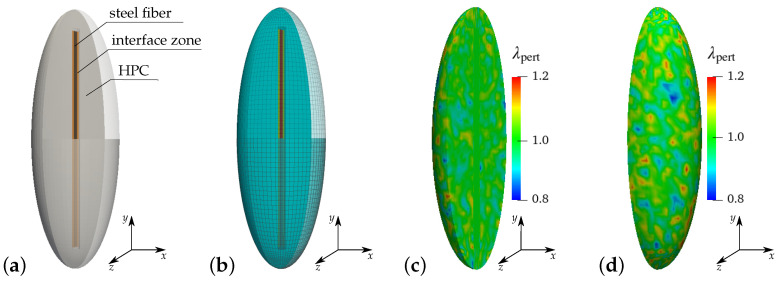
The representative volume element (RVE): (**a**) components of an ellipsoidal RVE and (**b**) a discretized ellipsoidal RVE used in the simulations, distribution of perturbation parameter $\lambda_{\mathrm{pert}}$ for the consideration of heterogeneity in the concrete phase of an ellipsoidal RVE, (**c**) a cross-sectional view and (**d**) a front view of the ellipsoidal RVE used in the simulations, adopted from [89].

The central element in the numerical homogenization for the analysis of a steel-fiber-reinforced HPC is the chosen ellipsoidal RVE. For the more general discussions on RVEs, see [86,87,88] and the references therein. The considered ellipsoidal RVE, consisting of the concrete matrix and a steel fiber, characterizes the material behavior along the preferred fiber direction. The components of this ellipsoidal unit cell are shown in Figure 8a. The ellipsoidal RVE is constructed considering that an embedded steel fiber occupies 0.3% of the total volume of the unit cell. The dimensions of the unit cell are selected to ensure that the volume fraction of fibers within the total volume of the RVE matches the fiber content specified for the concrete mixture. This corresponds to a fiber content of 23 kg/m^3^ in reinforced HPC. The ellipsoidal RVE containing a single steel fiber is discretized with 66,464 linear hexahedral elements, as shown in Figure 8b. The ellipsoidal RVE containing a single steel fiber is discretized with 63,232 linear hexahedral elements, as shown in Figure 8b.

**Figure 9:**
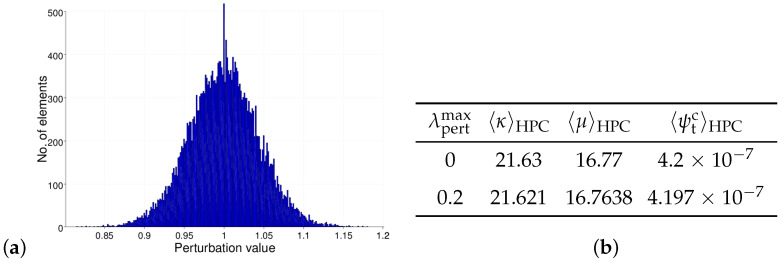
Consideration of inhomogeneity in the concrete phase of the ellipsoidal RVE: (**a**) histogram for 20% perturbation of material parameters of HPC and (**b**) comparison of the computed volume averages of perturbed material parameters of HPC, taken from [89].

The heterogeneity of the real concrete material influences the overall material behavior during failure. For this purpose, the effect of different percentages of heterogeneity on the failure behavior of concrete is studied in [89,90]. Following the same procedure, the heterogeneity in the concrete matrix is taken into account by implementing the random perturbation of the material parameters for an HPC matrix. The parameters $\kappa =\lambda_{\mathrm{pert}}\kappa_{0},\mu =\lambda_{\mathrm{pert}}\mu_{0}$ and $\psi^{c}=\lambda_{\mathrm{pert}}\psi_{0}^{c}$ are considered for perturbation. The perturbation parameter $\lambda_{\mathrm{pert}}$ is computed using the formula $\lambda_{\mathrm{pert}}=1+2\left(r_{\mathrm{pert}}-0.5\right)\lambda_{\mathrm{pert}}^{\max}$. Therein, the parameter $\lambda_{\mathrm{pert}}^{\max}$ controls the maximum percentage of perturbation and a random number $r_{\mathrm{pert}}\in R^{\left[0,1\right]}$ is used to achieve an individual random perturbation in each element. In this contribution, 20% perturbation of the material parameters is considered for all simulations. Thus, the value of parameters $\lambda_{\mathrm{pert}}^{\max}$ is taken as 0.2. The volume averages of the parameters, i.e., κ,μ and $\psi^{c}$, in the HPC phase yield values that are approximately equivalent to the unperturbed values of the corresponding parameters, i.e., $\kappa_{0}$, $\mu_{0}$ and $\psi_{0}^{c}$. The computed volume averages of parameters without perturbation and 20% perturbation are listed and compared in Figure 9b.

### 3.3. Failure Analysis of Pure and Reinforced HPC in Virtual Experiments 

For the analysis of the fundamental material behavior of an ellipsoidal RVE, six virtual experiments are considered. The presented micro-mechanical model of fracture based on the phase-field approach in Section 2 is used for the single-scale microscopic simulations of virtual experiments. For these simulations, a macroscopic homogeneous strain state is considered and applied to the microscopic boundary value problem based on the ellipsoidal RVE, see Figure 6. The boundary value problem for the virtual experiments consists of a cuboid with dimensions of 1×1×1 mm^3^ with different boundary conditions. Therein, an ellipsoidal RVE is attached at each integration point of the cuboid. Periodic boundary conditions are applied on the RVE at the microscale, see Section 3.1. The macroscopic boundary conditions are directly applied on the surface of the RVE by prescribing the macroscopic strain tensor $\bar{\varepsilon }$ as a homogeneous displacement of the boundary using $\bar{u}=\bar{\varepsilon }\;x$. The volumetric averages of the microscopic quantities over the RVE are calculated using Equation (21) to determine the corresponding macroscopic quantities. The virtual experiments are simulated using four different types of ellipsoidal RVEs, e.g., ellipsoidal RVEs of pure HPC with perturbation and without perturbation and ellipsoidal RVEs of reinforced HPC with perturbation and without perturbation of HPC parameters. The mechanical properties and the calibrated material parameters listed in Table 1 are used for the simulations of the RVEs on the microscale.

#### 3.3.1. Virtual Experiment I—Uniaxial Tensile Test with Vanishing Transverse Stresses

**Figure 10:**
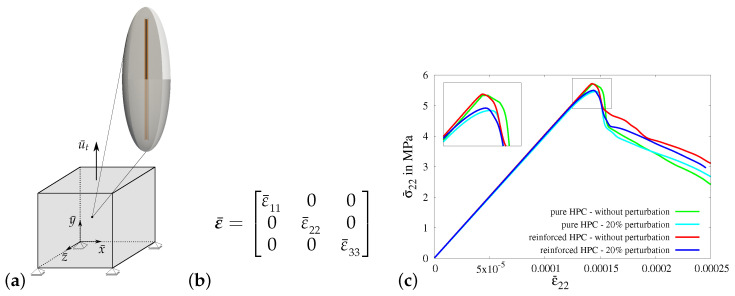
**Virtual experiment I**: uniaxial tensile test with vanishing transverse stresses. (**a**) Boundary value problem (RVE), (**b**) macroscopic strain tensor $\bar{\varepsilon }$ applied to the RVE and (**c**) comparison of macroscopic stress–strain characteristic using pure and reinforced ellipsoidal RVEs without perturbation and with 20% perturbation.

In virtual experiment I, a uniaxial tensile test (only ${\bar{\sigma }}_{22}\neq 0$) with vanishing transverse stresses is considered. The boundary value problem for virtual experiment I is shown in Figure 10a. The necessary boundary conditions are applied on the RVE by setting the value of strain in the *y*-direction ${\bar{\varepsilon }}_{22}$ using the macroscopic strain tensor $\bar{\varepsilon }$, see Figure 10b. The strains in the *x*-direction ${\bar{\varepsilon }}_{11}\left({\bar{\sigma }}_{11}=0\right)$ and in the *z*-direction ${\bar{\varepsilon }}_{33}\left({\bar{\sigma }}_{33}=0\right)$ are determined iteratively to achieve vanishing transverse stresses.

**Figure 11:**
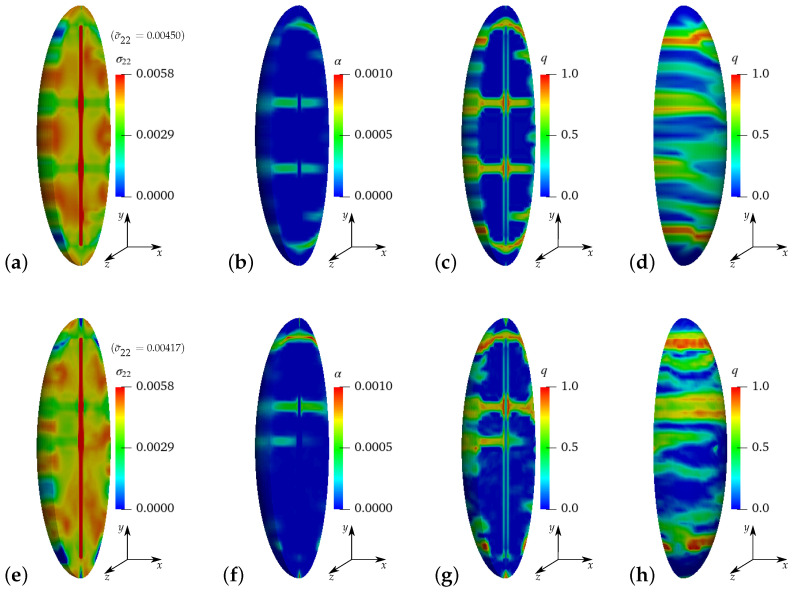
**Virtual experiment I**: uniaxial tensile test with vanishing transverse stresses using ellipsoidal RVEs of reinforced HPC: (**a**–**d**) HPC parameters without perturbation at ${\bar{\sigma }}_{22}=0.00450$ GPa and (**e**–**h**) with 20% perturbation at ${\bar{\sigma }}_{22}=0.00417$ GPa. Distribution of (**a**,**e**) microscopic stress $\sigma_{22}$ in GPa, (**b**,**f**) microscopic equivalent plastic strain α and microscopic phase-field parameter *q* in (**c**,**g**) a cross-sectional view and (**d**,**h**) a front view at a macroscopic strain of ${\bar{\varepsilon }}_{22}=0.175‰$.

In Figure 10c, the macroscopic stress–strain characteristics for virtual experiment I using all four types of RVEs are compared, i.e., the resulting macroscopic stress–strain curves for ellipsoidal RVEs of pure HPC without perturbation (green curve) and with perturbation (cyan curve) and ellipsoidal RVEs of reinforced HPC without perturbation (red curve) and with perturbation (blue curve). The comparison of these macroscopic responses shows that the reinforced HPC RVEs (blue and red curves) have higher macroscopic stresses in the *y*-direction at the peak than the corresponding pure HPC RVEs (green and cyan curves). The perturbed material parameters lower the tensile strength of the concrete material. This results in RVEs with perturbation (cyan and blue curves) failing earlier than the RVEs without perturbation (green and red curves), see Figure 11c.

The results of simulations of virtual experiment I using reinforced ellipsoidal RVEs without perturbation of HPC parameters at macroscopic stress ${\bar{\sigma }}_{22}=0.00450$ GPa, see Figure 11a–d, and with 20% perturbation of material parameters at macroscopic stress ${\bar{\sigma }}_{22}=0.00417$ GPa, see Figure 11e–h, are shown at a macroscopic strain of ${\bar{\varepsilon }}_{22}=0.175‰$. Therein, the distribution of microscopic stress $\sigma_{22}$ in the *y*-direction in GPa, see Figure 11a,e, the microscopic equivalent plastic strain α, see Figure 11b,f, and the microscopic phase-field parameter *q* in cross-sectional view, see Figure 11c,e, and in front view, see Figure 11d,h, can be compared to analyze the effect of the considered heterogeneity. For example, the phase-field parameter *q* in Figure 11g is not symmetrically distributed as compared to the distribution in Figure 11c.

#### 3.3.2. Numerical Results of Virtual Experiments II–V

In this section, the simulation results of virtual experiments II to V, focusing on the macroscopic stress–strain diagram, are compared and discussed. The plots comparing the distribution of various quantities over the RVE for all virtual experiments from II to V are included in the Appendix A.1.

*Virtual experiment II—transversely constrained uniaxial tensile test:*

**Figure 12:**
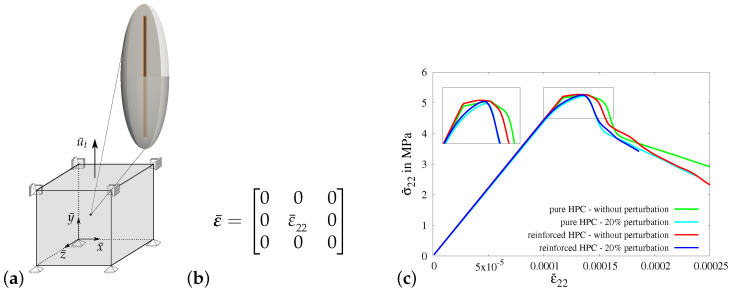
**Virtual experiment II**: a transversely constrained uniaxial tensile test: (**a**) boundary value problem (RVE), (**b**) macroscopic strain tensor $\bar{\varepsilon }$ applied to RVEs and (**c**) comparison of macroscopic stress–strain characteristics using pure and reinforced ellipsoidal RVEs without perturbation and with 20% perturbation.

In virtual experiment II, a uniaxial tensile test with a transversely constrained condition is considered. In contrast to the applied boundary conditions in virtual experiment I, the boundaries in the *x*- and *z*-directions are constrained in virtual experiment II, see Figure 12a. The boundary conditions are applied on the RVE by setting the value of strain in the *y*-direction ${\bar{\varepsilon }}_{22}$, see Figure 12b. The strains in the *x*-direction ${\bar{\varepsilon }}_{11}$ and in the *z*-direction ${\bar{\varepsilon }}_{33}$ are set to zero to achieve a transversely constrained condition. In Figure 12c, the macroscopic stress–strain characteristics for virtual experiment II using all four types of RVEs are plotted. The comparison of the macroscopic results for virtual experiment II shows similar observations, as found in the comparison of the resulting curves for virtual experiment I.

*Virtual experiment III—uniaxial tensile test with transverse compression:*

**Figure 13:**
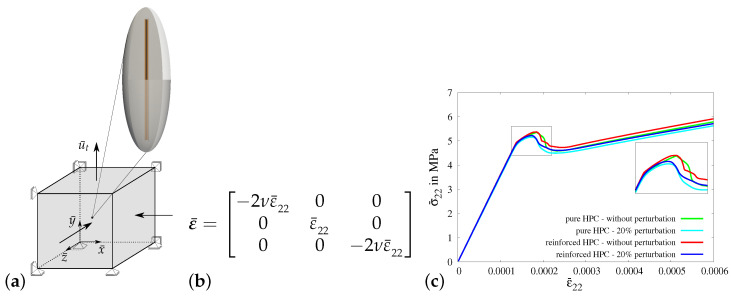
**Virtual experiment III**: uniaxial tensile test with transverse compression: (**a**) boundary value problem (RVE), (**b**) macroscopic strain tensor $\bar{\varepsilon }$ applied to RVE and (**c**) comparison of macroscopic stress–strain characteristics using pure and reinforced ellipsoidal RVE without perturbation and with 20% perturbation.

In virtual experiment III, a uniaxial tensile test with transverse compression is considered. The boundary value problem for virtual experiment III is shown in Figure 13a. The boundary conditions are applied on the RVE by setting the value of macroscopic strain in the *y*-direction ${\bar{\varepsilon }}_{22}$, see Figure 13b. The strains in the *x*-direction ${\bar{\varepsilon }}_{11}$ and in the *z*-direction ${\bar{\varepsilon }}_{33}$ are set to ${\bar{\varepsilon }}_{11}={\bar{\varepsilon }}_{33}=-2\;\nu \;{\bar{\varepsilon }}_{22}$ to achieve the condition of transverse compression in the *x*- and *z*-directions. In Figure 13c, the macroscopic stress–strain characteristics for virtual experiment III using all four types of RVEs are plotted. The comparison of the resulting curves for virtual experiment III shows similar observations to those found in the cases of virtual experiments I and II. The macroscopic stress in the *y*-direction in the reinforced HPC RVEs (red and blue curves) and the RVEs without perturbation (green and red curves) is higher than that in the pure HPC RVEs (green and cyan curves) and RVEs with perturbation (cyan and blue curves), respectively, see Figure 13c. However, the applied transverse compression delays complete failure, resulting in an increase in the maximum load bearing capacity of the concrete material, see Figure 13c.

*Virtual experiment IV—uniaxial tensile test in the transverse direction:*

**Figure 14:**
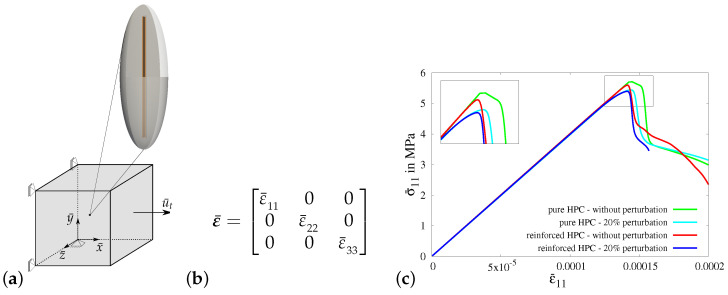
**Virtual experiment IV**: uniaxial tensile test in the transverse direction: (**a**) boundary value problem (RVE), (**b**) macroscopic strain tensor $\bar{\varepsilon }$ applied to RVEs and (**c**) comparison of macroscopic stress–strain characteristics using pure and reinforced ellipsoidal RVEs without perturbation and with 20% perturbation.

In virtual experiment IV, a uniaxial tensile test (only ${\bar{\sigma }}_{11}\neq 0$) in the transverse direction is considered. The boundary value problem for virtual experiment IV is shown in Figure 13a. The load is applied along the *x*-direction, which is perpendicular to the preferred fiber direction according to the alignment of the RVE, as shown in Figure 14a. The boundary conditions are applied on the RVE by setting the value of strain in the *x*-direction ${\bar{\varepsilon }}_{11}$, see Figure 14b. The strains in the *y*-direction ${\bar{\varepsilon }}_{22}\left({\bar{\sigma }}_{22}=0\right)$ and in the *z*-direction ${\bar{\varepsilon }}_{33}\left({\bar{\sigma }}_{33}=0\right)$ are determined iteratively to ensure no transverse stresses.

In Figure 14c, the macroscopic stress–strain characteristics for virtual experiment IV using all four types of RVEs are plotted. The resulting curves show the same characteristics in the simulations using all RVEs before the start of the evolution of damage, i.e.,before evolution of phase-field parameter *q*. However, the RVEs with perturbation (cyan and blue curves) fail earlier than the RVEs without perturbation (green and red curves), see Figure 14c. This is because of the perturbation of parameters, especially the perturbation of the critical fracture energy in tension $\psi_{t}^{c}$. On the contrary to the stress–strain characteristics for virtual experiment I, II and III, the macroscopic responses of the reinforced HPC RVEs (red and blue curves) have lower macroscopic stress in the *x*-direction at the peak than that in the pure HPC RVEs (green and cyan curves). Here, the RVEs without perturbation (green curve with red curve) and the RVEs with perturbation (cyan curve with blue curve) are compared. This shows that there is no additional stiffness in the concrete material in the direction transverse to the fiber. In fact, the additional material at the fiber–concrete interface is a potential location for crack initiation.

*Virtual experiment V—shear test:*

**Figure 15:**
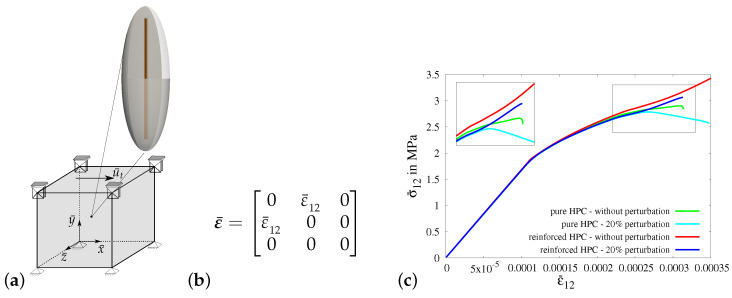
**Virtual experiment V**: shear test. (**a**) Boundary value problem (RVE), (**b**) macroscopic strain tensor $\bar{\varepsilon }$ applied to RVE and (**c**) comparison of macroscopic stress–strain characteristics using pure and reinforced ellipsoidal RVEs without perturbation and with 20% perturbation.

In virtual experiment V, a shear test is considered, see Figure 15a. The boundary value problem for virtual experiment IV is shown in Figure 15a. The boundary conditions are applied on the RVE by setting the same value for the shear strains ${\bar{\varepsilon }}_{12}$ and ${\bar{\varepsilon }}_{21}$, see Figure 15b. In Figure 15c, the macroscopic stress–strain characteristics for virtual experiment IV using all four types of RVEs are plotted. The resulting curves initially follow the same stress–strain characteristics but later, they deviate from each other. The macroscopic stress ${\bar{\sigma }}_{12}$ in the reinforced HPC RVEs (blue and red curves) is higher than that in the pure HPC RVEs (green and cyan curves), see Figure 15c. The resulting curves for the RVEs with perturbation (cyan and blue curves) lie below the resulting curves for RVEs without perturbation (green and red curves), see Figure 15c.

## 4. Phenomenological Material Model for Fiber-Reinforced HPC

### 4.1. Constitutive Framework

Fiber-reinforced HPC is a mixture of components, i.e., the HPC matrix and the embedded steel fibers, which makes reinforced HPC heterogeneous. Thus, for the representation of steel-fiber-reinforced HPC, an additive structure of the macroscopic stored energy function per unit volume ψ is formulated, cf. [90], i.e.,

(25) $$\psi =v^{\mathrm{HPC}}\;\psi^{\mathrm{HPC}}\left(\varepsilon ,\varepsilon^{p,\mathrm{HPC}},q,\nabla q,\alpha^{\mathrm{HPC}}\right)+v^{F}\;\psi^{F}\left(\varepsilon ,M,\alpha^{\mathrm{HPC}}\right)\;.$$

where the conservation of the volume fraction $v^{\mathrm{HPC}}$ of the HPC phase and the volume fraction $v^{F}$ of fibers is ensured by the condition $v^{\mathrm{HPC}}=1-v^{F}$.

It is observed in the experiments that fracture occurs only in the HPC phase. Therefore, the phase-field parameter q∈[0,1] at the macroscopic level is considered active only in the HPC phase describing the damage therein. An energy function $\psi^{\mathrm{HPC}}$ with a similar structure to the energy function used in the micro-mechanical model, see Equation (9), is constructed for the description of fracture in the HPC phase:

(26) $$\psi^{\mathrm{HPC}}=g\left(q\right)\left[\psi_{0}^{e+,\mathrm{HPC}}+\psi_{0}^{p,\mathrm{HPC}}-\psi^{c,\mathrm{HPC}}\right]+\psi_{0}^{e-,\mathrm{HPC}}+\psi^{c,\mathrm{HPC}}\left[1+\frac{q^{2}+l^{2}{\left||\nabla q|\right|}^{2}}{\zeta }\right]\;,$$

where ∇q denotes the gradient of the phase-field parameter *q* and $g\left(q,m\right)={\left(1-q\right)}^{m}$ is the considered degradation function, cf. [67,68]. The specific critical fracture energy $\psi^{c,\mathrm{HPC}}$, the length-scale parameter *l* and the parameters *m* and ζ play the same role, as described in Section 2, see Equation (9). The reference elastic energy function $\psi_{0}^{e}\left(\varepsilon^{e,\mathrm{HPC}}\right)$ is additively decomposed into a positive $\psi_{0}^{e+}\left(\varepsilon^{e,\mathrm{HPC}}\right)$ and a negative part $\psi_{0}^{e-}\left(\varepsilon^{e,\mathrm{HPC}}\right)$, respectively, as

(27) $$\begin{array}{cc} \; & \psi_{0}^{e+,\mathrm{HPC}}\left(\varepsilon^{e,\mathrm{HPC}}\right)=\kappa {\langle \mathrm{tr}\left[\varepsilon^{e,\mathrm{HPC}}\right]\rangle }_{+}^{2}/2+\mu ||\mathrm{dev}\varepsilon^{e,\mathrm{HPC}}{\left|\right|}^{2}\;,\\ \; & \psi_{0}^{e-,\mathrm{HPC}}\left(\varepsilon^{e,\mathrm{HPC}}\right)=\kappa {\langle \mathrm{tr}\left[\varepsilon^{e,\mathrm{HPC}}\right]\rangle }_{-}^{2}/2\;, \end{array}$$

where μ and κ are the shear and bulk modulus of the HPC phase and ${\langle •\rangle }_{\pm }=1/2\;\left(\;•\pm |•|\;\right)$ is Macaulay’s notation. The elastic strain tensor $\varepsilon^{e,\mathrm{HPC}}$ for the HPC phase is defined by

(28) $$\varepsilon^{e,\mathrm{HPC}}:=\varepsilon -\varepsilon^{p,\mathrm{HPC}}\;,$$

using a plastic strain tensor $\varepsilon^{p,\mathrm{HPC}}$ and a total macroscopic strain tensor ε for the HPC phase, i.e.,

(29) $$\varepsilon =\nabla_{s}u=\frac{1}{2}\left(\nabla_{s}u+\nabla_{s}^{T}u\right)\;.$$

The plastic energy $\psi^{p,\mathrm{HPC}}$ for the HPC phase is considered as

(30) $$\psi^{p,\mathrm{HPC}}\left(\alpha^{\mathrm{HPC}}\right)={\left(1-q\right)}^{m}\psi_{0}^{p,\mathrm{HPC}}\;\mathrm{with}\;\psi_{0}^{p,\mathrm{HPC}}=\left[y_{0}^{\mathrm{HPC}}\;\alpha^{\mathrm{HPC}}+\frac{1}{2}\;h^{\mathrm{HPC}}{\left(\alpha^{\mathrm{HPC}}\right)}^{2}\right]\;,$$

where $y_{0}^{\mathrm{HPC}}$ is the yield stress and $h^{\mathrm{HPC}}$ is the hardening parameter for the HPC phase. The equivalent plastic strain for the HPC phase is denoted by $\alpha^{\mathrm{HPC}}$. The energy function $\psi^{F}$, which describes the behavior of the embedded steel fiber aligned in the preferred direction a, is

(31) $$\psi^{F}\left(\varepsilon ,M,e^{F},e^{p,F},\alpha^{F}\right)=\psi^{e,F}\left(\varepsilon ,M,e^{F},e^{p,F}\right)+\psi^{p,F}\left(\alpha^{F}\right)\;,$$

which is governed by the structural tensor M:=a⊗a using the preferred direction of fiber a with the property that ||a||=1. Therein, the one-dimensional elastic–plastic problem is considered, which represents the elasto-plasticity due to an embedded steel fiber oriented in the preferred direction a. Accordingly, the elastic energy function $\psi^{e,F}$ for the fibers is formulated as

(32) $$\psi^{e,F}\left(\varepsilon ,M,e^{F},e^{p,F}\right)=\frac{1}{2}\;E^{F}{\left(e^{e,F}\right)}^{2}\;,$$

where $E^{F}$ is the elastic moduli of the fibers. The total strain tensor $e^{F}$ for the fibers is defined as

(33) $$e^{F}=\varepsilon :M\;.$$

The elastic strain tensor $e^{e,F}$ for the fiber can be calculated using the plastic strain $e^{p,F}$ and the total strain $e^{F}$ of the fibers, i.e.,

(34) $$e^{e,F}=e^{F}-e^{p,F}\;.$$

The plastic energy $\psi^{p,F}$, depending on the equivalent plastic strain $\alpha^{F}$ of the fibers, is

(35) $$\psi^{p,F}\left(\alpha^{F}\right)=y_{0}^{F}\;\alpha^{F}+\frac{1}{2}\;h^{\mathrm{HPC}}\;{\left(\alpha^{F}\right)}^{2}\;,$$

where $y_{0}^{F}$ and $h^{F}$ denote the yield stress and the hardening parameter for the fibers, respectively. The additive structure for the macroscopic stress tensor is defined as

(36) $$\sigma =v^{\mathrm{HPC}}\;\sigma^{\mathrm{HPC}}+v^{F}\;\sigma^{F}\;,$$

where the stress tensor $\sigma^{\mathrm{HPC}}$ for the HPC phase is computed by

(37) $$\begin{array}{cc} \; & \sigma^{\mathrm{HPC}}={\left(1-q\right)}^{m}\underset{\sigma_{0}^{+,\mathrm{HPC}}}{\underset{︸}{\left[\mathrm{tr}\;\sigma_{0}^{+,\mathrm{HPC}}\;I+\mathrm{dev}\sigma_{0}^{\mathrm{HPC}}\right]}}+\underset{\sigma_{0}^{-,\mathrm{HPC}}}{\underset{︸}{\left[\mathrm{tr}\;\sigma_{0}^{+,\mathrm{HPC}}\;I\right]}}\;, \end{array}$$

(38) $$\begin{array}{cc} \; & \mathrm{with}\;\mathrm{tr}\;\sigma_{0}^{+,\mathrm{HPC}}=\kappa {\langle \mathrm{tr}\;\varepsilon^{e,\mathrm{HPC}}\rangle }_{+},\;\mathrm{dev}\sigma_{0}^{\mathrm{HPC}}=2\mu \;\mathrm{dev}\varepsilon^{e,\mathrm{HPC}}\;, \end{array}$$

(39) $$\begin{array}{cc} \; & \mathrm{and}\;\mathrm{tr}\;\sigma_{0}^{-,\mathrm{HPC}}=\kappa {\langle \mathrm{tr}\;\varepsilon^{e,\mathrm{HPC}}\rangle }_{-}\;. \end{array}$$

Therein, $\sigma_{0}^{\mathrm{HPC}}=\sigma_{0}^{+,\mathrm{HPC}}+\sigma_{0}^{-,\mathrm{HPC}}$ is the effective stress tensor for the HPC phase. The stress tensor $\sigma^{F}$ for the fibers is defined as

(40) $$\sigma^{F}=\sigma^{F}\left(a⊗a\right)\;\mathrm{with}\;\sigma^{F}=E^{F}\left(e^{F}-e^{p,F}\right)\;.$$

The governing equation for the phase-field parameter *q* is computed using the macroscopic stored energy function for the HPC phase $\psi^{\mathrm{HPC}}$, see Equation (26), as

(41) $$q-l^{2}\;\mathrm{Div}\left(\nabla q\right)-\left(1-q\right)H^{\mathrm{HPC}}=0\;,$$

where, to ensure the upper and lower bounds of the range of the phase-field parameter q∈[0,1], the parameter m=2 is set. Herein, a local history field $H^{\mathrm{HPC}}$ is constructed to ensure the irreversibility of the crack evolution in the HPC phase, i.e.,

(42) $$H^{\mathrm{HPC}}:=\max_{s\in [0,t]}\;H_{0}^{\mathrm{HPC}}\left(x,t\right)\geq 0\;\mathrm{with}\;H_{0}^{\mathrm{HPC}}=\zeta \langle \left[\frac{\psi_{0}^{e+,\mathrm{HPC}}+\psi_{0}^{p,\mathrm{HPC}}}{\psi^{c,\mathrm{HPC}}}\right]-1\rangle \;.$$

where the maximum value of a dimensionless crack driving state function $H_{0}^{\mathrm{HPC}}$ for the HPC phase is considered and its contribution to the evolution of the phase-field parameter *q* is weighted by the volume fraction $v^{\mathrm{HPC}}$ of the HPC phase. During the loading process, the local history field is updated according to

(43) $$\begin{array}{c} H^{\mathrm{HPC}}=\left\{\begin{array}{c} H_{0,n+1}^{\mathrm{HPC}}\;\;\;\mathrm{for}\;\;H_{0,n+1}^{\mathrm{HPC}}>H_{0,n}^{\mathrm{HPC}}\;,\\ H_{0,n}^{\mathrm{HPC}}\;\;\;\;\;\mathrm{otherwise}\;. \end{array}\right. \end{array}$$

The distinct behavior of concrete under tension and in compression is modeled by using two parameters for the critical fracture energy in tension $\psi_{t}^{c}$ and in compression $\psi_{c}^{c}$. These parameters are distinguished from each other based on the sign of the first invariant of the stress tensor using the condition:

(44) $$\begin{array}{c} \psi^{c}=\left\{\begin{array}{c} \psi_{t}^{c},\;\mathrm{for}\;\;\mathrm{tr}\;\varepsilon \geq 0\;,\\ \psi_{c}^{c},\;\mathrm{otherwise}\;. \end{array}\right. \end{array}$$

#### 4.1.1. Yield Criteria and Flow Rules

The nonlinear behavior of reinforced HPC is mainly characterized by the combination of the material behaviors of concrete and steel fibers. To capture the nonlinearity due to elasto-plasticity in the HPC matrix and the steel fibers, two separate yield criteria are used. The first criterion is the associative Drucker–Prager yield plasticity which is considered for the HPC phase, cf. [74], i.e.,

(45) $$\phi^{\mathrm{HPC}}\left(\sigma_{0}^{\mathrm{HPC}},\kappa_{p}^{\mathrm{HPC}}\right)=\frac{1}{\sqrt{2}}\;||\mathrm{dev}\sigma^{\mathrm{HPC}}\left|\right|-\beta_{p}^{\mathrm{HPC}}\mathrm{tr}\sigma_{0}^{\mathrm{HPC}}-\kappa_{p}^{\mathrm{HPC}}\;,$$

where $\beta_{p}^{\mathrm{HPC}}$ is the coefficient of the hydrostatic stress component and $\kappa_{p}$ denotes the hardening function for the HPC phase:

(46) $$\kappa_{p}^{\mathrm{HPC}}:=\partial_{\alpha }\psi_{0}^{p,\mathrm{HPC}}=y_{0}^{\mathrm{HPC}}+h^{\mathrm{HPC}}\alpha^{\mathrm{HPC}}\;.$$

The plastic behavior associated with the given yield criterion is dependent on the effective stress tensor, $\sigma_{0}^{\mathrm{HPC}}=\sigma_{0}^{+,\mathrm{HPC}}+\sigma_{0}^{-,\mathrm{HPC}}$, see Equation (39). The rate of plastic strain ${\dot{\varepsilon }}^{p,\mathrm{HPC}}$ and the equivalent plastic strain ${\dot{\alpha }}^{\mathrm{HPC}}$ are, respectively, given as,

(47) $${\dot{\varepsilon }}^{p,\mathrm{HPC}}=\lambda^{p,\mathrm{HPC}}\;\frac{\partial \phi^{\mathrm{HPC}}}{\partial \sigma_{0}^{\mathrm{HPC}}}=\lambda^{p,\mathrm{HPC}}\;n_{\mathrm{DP}}^{\mathrm{HPC}}\;\mathrm{and}\;{\dot{\alpha }}^{\mathrm{HPC}}=-\lambda^{p,\mathrm{HPC}}\;\frac{\partial \phi^{\mathrm{HPC}}}{\partial \kappa_{p}^{\mathrm{HPC}}}=\lambda^{p,\mathrm{HPC}}\;,$$

where $\lambda^{p,\mathrm{HPC}}$ denotes the incremental plastic multiplier. The unit normal $n_{\mathrm{DP}}^{\mathrm{HPC}}$ on the yield surface for the HPC phase is

(48) $$n_{\mathrm{DP}}^{\mathrm{HPC}}=\frac{\partial \phi^{\mathrm{HPC}}}{\partial \sigma_{0}^{\mathrm{HPC}}}=\frac{1}{\sqrt{2}}\;n^{\mathrm{HPC}}+\beta_{p}I\;\mathrm{with}\;n_{\mathrm{DP}}^{\mathrm{HPC}}=\frac{\mathrm{dev}\sigma_{0}^{\mathrm{HPC}}}{||\mathrm{dev}\sigma_{0}^{\mathrm{HPC}}\left|\right|}\;.$$

The considered Kuhn–Tucker criterion is given as

(49) $$\phi^{\mathrm{HPC}}\left(\sigma_{0}^{\mathrm{HPC}},\kappa_{p}^{\mathrm{HPC}}\right)\leq 0\;,\;\;\lambda^{p,\mathrm{HPC}}\geq 0\;,\;\;\lambda^{p,\mathrm{HPC}}\phi^{\mathrm{HPC}}\left(\sigma_{0}^{\mathrm{HPC}},\kappa_{p}^{\mathrm{HPC}}\right)=0\;.$$

To capture nonlinear behavior along the preferred fiber direction, a one-dimensional von Mises yield criterion is used, cf. [75,91], i.e.,

(50) $$\phi^{F}\left(\sigma^{F},\kappa_{p}^{F}\right)=\left|\sigma^{F}\right|-\kappa_{p}^{F}\;\mathrm{with}\;\kappa_{p}^{F}:=\partial_{\alpha }\psi^{p,F}=y_{0}^{F}+h^{F}\alpha^{F}\;,$$

The rate of plastic strain ${\dot{e}}^{p,F}$ and the equivalent plastic strain ${\dot{\alpha }}^{F}$ for the fibers are

(51) $${\dot{e}}^{p\;F}=\lambda^{p,F}\;\mathrm{sign}\left(\sigma \right)\;\mathrm{and}\;{\dot{\alpha }}^{F}=-\lambda^{p,F}\;\frac{\partial \phi^{F}}{\partial \kappa_{p}^{F}}=\lambda^{p,F}\;,$$

where $\lambda^{p,F}$ denotes the incremental plastic multiplier with the Kuhn–Tucker criteria, which are

(52) $$\phi^{F}\left(\sigma_{0}^{F},\kappa_{p}^{F}\right)\leq 0\;,\;\;\lambda^{p,F}\geq 0\;,\;\;\lambda^{p,F}\phi^{F}\left(\sigma_{0}^{F},\kappa_{p}^{F}\right)=0\;.$$

#### 4.1.2. Consistent Elasto-Plastic Tangent Moduli

Nonlinear systems are solved iteratively using the elastic predictor–plastic corrector method (radial-return mapping) for the Drucker–Prager plasticity, see [70], and for further details, see [91,92]. Therefore, we define the trial elastic strains in the HPC phase and in the fibers as

(53) $$\varepsilon_{n+1}^{e^{\mathrm{tr}},\mathrm{HPC}}=\varepsilon_{n+1}-\varepsilon_{n}^{p^{\mathrm{tr}},\mathrm{HPC}}\;\mathrm{and}\;e_{n+1}^{e^{\mathrm{tr}},F}=e_{n+1}^{F}-e_{n}^{p^{\mathrm{tr}},F}\;.$$

**Figure 16:**
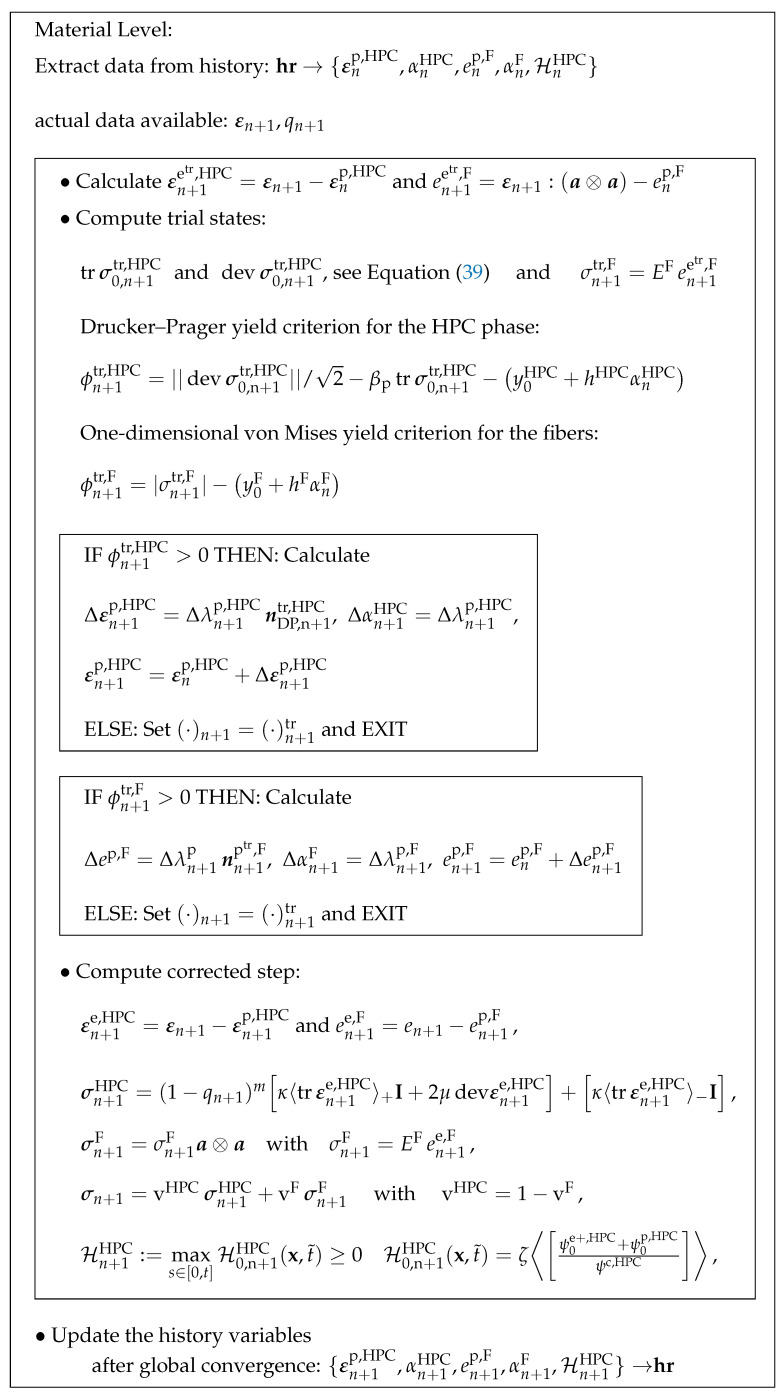
Elasto-plastic return algorithm.

Therein, a trial quantity is denoted by ${\left(\cdot \right)}^{\mathrm{tr}}$ and *n* and n+1 denote the values at time $t_{n}$ and $t_{n+1}$, respectively. The stress update is explained in Figure 16. The volumetric part $C_{\mathrm{vol},n+1}^{e,\mathrm{HPC}}$ and deviatoric part $C_{\mathrm{dev},n+1}^{e^{,}\mathrm{HPC}}$ of the elastic tangent moduli $C_{n+1}^{e,\mathrm{HPC}}$ are defined by

(54) $$C_{\mathrm{vol},n+1}^{e,\mathrm{HPC}}=\kappa \;I⊗I\;\mathrm{and}\;C_{\mathrm{dev},n+1}^{e,\mathrm{HPC}}=2\;\mu \;I\;P\;\mathrm{with}\;I\;P=I\;I-\frac{1}{3}\;I⊗I\;,$$

where I is the second-order identity tensor and II denotes the fourth-order symmetric identity tensor. The elastic tangent moduli $C_{n+1}^{e,\mathrm{HPC}}$ is formulated by following the additive split of the reference energy function based on the sign of the first invariant, see Equation (27), cf. [70,93], as

(55) $$\begin{array}{c} C_{n+1}^{e,\mathrm{HPC}}=v^{\mathrm{HPC}}\left\{\begin{array}{cc} g\left(q,m\right)\left[C_{\mathrm{vol},n+1}^{e,\mathrm{HPC}}+C_{\mathrm{dev},n+1}^{e,\mathrm{HPC}}\right] & \mathrm{if}\;\;\mathrm{tr}\varepsilon_{n+1}^{e^{\mathrm{tr}},\mathrm{HPC}}\geq 0\;,\\ \left[C_{\mathrm{vol},n+1}^{e,\mathrm{HPC}}+g\left(q,m\right)\;C_{\mathrm{dev},n+1}^{e,\mathrm{HPC}}\right] & \mathrm{if}\;\;\mathrm{tr}\varepsilon_{n+1}^{e^{\mathrm{tr}},\mathrm{HPC}}<0\;. \end{array}\right. \end{array}$$

Similarly, the elastic tangent modulus in the preferred fiber direction is computed as

(56) $$C_{n+1}^{e,F}=v^{F}E^{F}\left(a⊗a⊗a⊗a\right)\;.$$

In the elastic–plastic case, return mapping for plastic correction is necessary. For this purpose, Equations (47) and (51) are solved while satisfying the yield criteria, i.e., Equations (45) and (50), at the discrete time step $t_{n+1}$, see Figure 16. The volumetric part $C_{\mathrm{vol},n+1}^{\mathrm{ep},\mathrm{HPC}}$ and deviatoric part $C_{\mathrm{dev},n+1}^{\mathrm{ep},\mathrm{HPC}}$ of the elasto-plastic tangent modulus $C_{n+1}^{\mathrm{ep},\mathrm{HPC}}$ can be derived using return mapping, see [92,94]. This follows

(57) $$\begin{array}{cc} \; & C_{\mathrm{vol},n+1}^{\mathrm{ep},\mathrm{HPC}}=\kappa \;I⊗I-3\kappa \beta_{p}I⊗D\;, \end{array}$$

(58) $$\begin{array}{cc} \; & C_{\mathrm{dev},n+1}^{\mathrm{ep},\mathrm{HPC}}=2\;\mu \;\delta_{1}I\;P+2\;\mu \left(1-\delta_{1}\right)\;n_{n+1}^{\mathrm{tr},\mathrm{HPC}}⊗n_{n+1}^{\mathrm{tr},\mathrm{HPC}}-\sqrt{2}\;\mu \;n_{n+1}^{\mathrm{tr},\mathrm{HPC}}⊗D\;, \end{array}$$

where the second-order tensor D and factor $\delta_{1}$ are given by

(59) $$D=\frac{3\kappa \beta_{p}I+\sqrt{2}\;\mu \;n_{n+1}^{\mathrm{tr},\mathrm{HPC}}}{\mu +9\beta_{p}^{2}\;\kappa +h^{\mathrm{HPC}}}\;\mathrm{and}\;\delta_{1}=1-\frac{\sqrt{2}\;\mu \;\Delta \lambda_{n+1}^{p,\mathrm{HPC}}}{||\mathrm{dev}\sigma_{0,n+1}^{\mathrm{tr},\mathrm{HPC}}\left|\right|}\;,$$

where $\Delta \lambda_{n+1}^{p,\mathrm{HPC}}:=\lambda_{n+1}^{p,\mathrm{HPC}}\left(t_{n+1}-t_{n}\right)$ denotes the incremental Lagrange multiplier, see [70]. The consistent elasto-plastic tangent modulus $C_{n+1}^{\mathrm{ep},\mathrm{HPC}}$ appears, cf. [93],

(60) $$\begin{array}{c} C_{n+1}^{\mathrm{ep},\mathrm{HPC}}=v^{\mathrm{HPC}}\left\{\begin{array}{cc} g\left(q,m\right)\left[C_{\mathrm{vol},n+1}^{\mathrm{ep},\mathrm{HPC}}+C_{\mathrm{dev},n+1}^{\mathrm{ep},\mathrm{HPC}}\right], & \mathrm{if}\;\;\mathrm{tr}\varepsilon_{n+1}^{e^{\mathrm{tr}},\mathrm{HPC}}\geq 0\;,\\ \left[C_{\mathrm{vol},n+1}^{\mathrm{ep},\mathrm{HPC}}+g\left(q,m\right)\;C_{\mathrm{dev},n+1}^{\mathrm{ep},\mathrm{HPC}}\right], & \mathrm{if}\;\;\mathrm{tr}\varepsilon_{n+1}^{e^{\mathrm{tr}},\mathrm{HPC}}<0\;. \end{array}\right. \end{array}$$

The consistent elasto-plastic tangent modulus $C_{n+1}^{\mathrm{ep},F}$ for the fibers is given by

(61) $$C_{n+1}^{\mathrm{ep},F}=\frac{v^{F}E^{F}h^{F}}{E^{F}+h^{F}}\left(a⊗a⊗a⊗a\right)\;.$$

It is important to note that in the described formulation of the phenomenological material model, plastic flow takes place independently in both the HPC phase and the fibers. Consequently, the overall consistent elasto-plastic tangent modulus can be obtained through the additive combination of Equations (54), (56), (60), and (61). The specific nature of this additive combination depends on whether elasticity or elasto-plasticity occurs in each individual phase. The weak formulation of the balance of linear momentum, i.e., Equation (17) using the total stress tensor, see Equation (36), and of the governing equation for the phase-field parameter *q*, i.e., Equation (18), using a local history field $H^{\mathrm{HPC}}$, see Equations (41) and (42), is solved using the framework of the Finite Element Method, see [76]. The supplementary data for the FE analysis of macroscopic BVPs are provided in [95]. An algorithm for the implementation of the formulations in the presented model is explained in Figure 16.

### 4.2. Numerical Calibration and Validation Using Unit Cell Calculations

In this section, numerical simulations using the presented phenomenological material model on a single-scale are documented. Simulations are conducted for macroscopic boundary value problems (BVPs), which involves cuboids made of both pure and reinforced HPC. The same homogeneous boundary conditions as those applied in the simulations of virtual experiments using RVEs in Section 3.3 are used for the simulations. The dimensions of the cuboid, i.e., length and width, are selected to match the maximum measurements of the ellipsoidal RVE. This ensures that the cuboid can accommodate an ellipsoidal RVE entirely within its boundaries, e.g., see Figure 19a.

The fiber exhibits the same preferred direction within both the cuboid and the constructed ellipsoidal RVE, i.e., aligning parallel to the *y*-axis, see Figure 19a. Note that for the simulations of macroscopic BVPs, the orientation of the fiber within the cuboid is set using the preferred fiber direction a. In the cuboid-based simulations, the material parameters used for the HPC phase and fibers are identical to the material parameters utilized in the virtual experiment simulations, as listed in Table 2. The fiber volume fraction is $v^{F}=0.003$, corresponding to a fiber content of 23 kg/m^3^ within the reinforced HPC.

#### 4.2.1. Sensitivity Analysis for the Calibration of the Length-Scale Parameter *l*

The calibration of the length-scale parameter *l* at the macroscale is necessary due to a shift in the scale, i.e., from the microscale to macroscale, which is accomplished through a comparison of the macroscopic stress–strain curves obtained from simulations of macroscopic BVP I, see Figure 19a, and virtual experiments I, see Figure 10a, for pure HPC without any perturbations.

**Figure 17:**
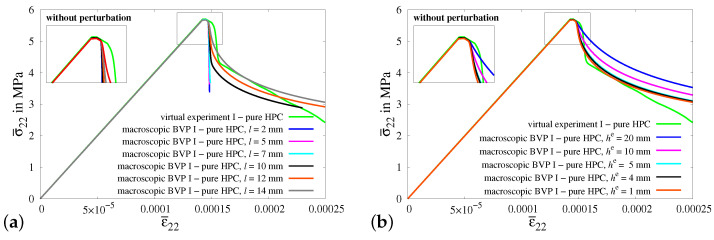
Comparison of macroscopic stress–strain characteristics with the results of uniaxial tension tests performed in virtual experiment I using an ellipsoidal RVE and a phenomenological material model applied to the macroscopic BVP I (pure HPC without perturbations). (**a**) Calibration of length-scale parameter *l* using $h^{e}=1$ mm and (**b**) convergence study using various element sizes $h^{e}$ using l=14 mm.

For the numerical analysis of macroscopic BVP I, homogeneous boundary conditions representing a uniaxial tensile test without transverse stresses are applied on a cuboid of pure HPC. Initially, the simulations of macroscopic BVP I are conducted using various values of the length-scale parameter *l*, while maintaining a uniform mesh size of $h^{e}=1$ mm. The cuboid is discretized for the simulations using 24,000 linear hexahedral elements. The value of the length-scale parameter l=14 mm corresponding to the most accurately fitted stress–strain curve from the simulations of macroscopic BVP I is selected, see Figure 17a. Furthermore, a convergence study is conducted using various element sizes $h^{e}$ using the calibrated value of the length-scale parameter l=14 mm. Variations in the mesh density (coarsening) were applied in order to check the convergence with respect to the mesh density. The macroscopic BVP I is simulated using various mesh sizes as part of the regularization study based on the length-scale parameter l=14 mm, see Figure 17b. Based on the comparison of results, a length-scale parameter of l=14 mm and a mesh size of $h^{e}=1$ mm are selected for all the subsequent simulations of macroscopic BVPs.

#### 4.2.2. Macroscopic BVP I—Uniaxial Tensile Test with Vanishing Transverse Stresses

**Figure 18:**
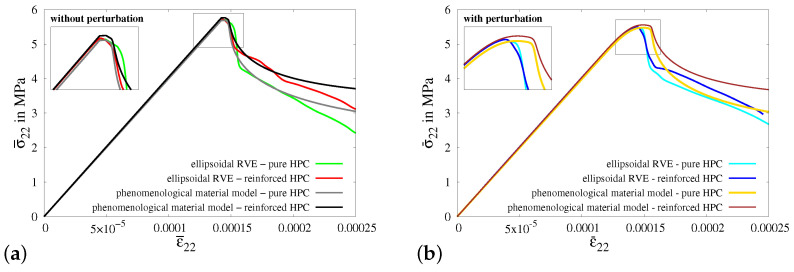
**Macroscopic BVP I**: comparison of macroscopic stress–strain characteristics with the results of virtual experiment I using ellipsoidal RVEs (**a**) without perturbation and (**b**) with 20% perturbation of HPC parameters.

The resulting stress–strain plots obtained from simulations of virtual experiment I and macroscopic BVP I without and with 20% perturbations of HPC parameters are compared in Figures 18a and  18b, respectively. In Figure 18a, the stress–strain curve calibrated for a pure HPC cuboid in macroscopic BVP I (gray curve) and the resultant curve obtained for the pure HPC RVE in virtual experiment I (green curve) are taken from Figure 17.

**Figure 19:**
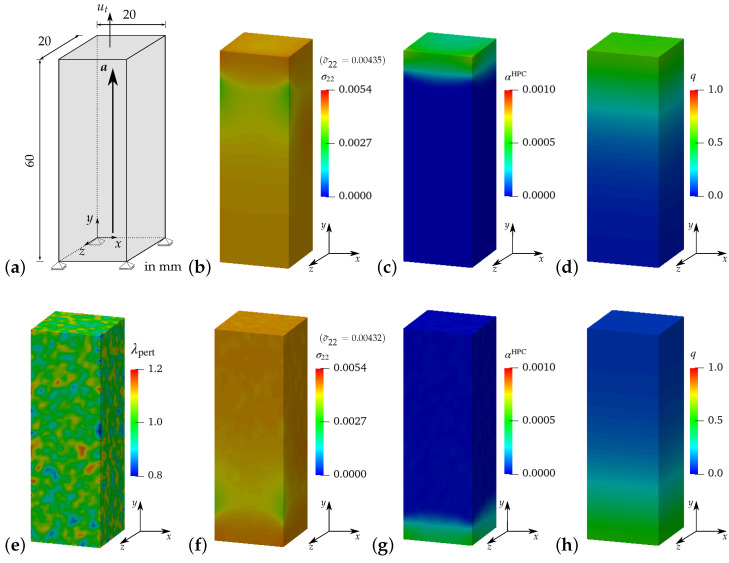
Phenomenological material model applied to (**a**) **macroscopic BVP I**, (**b**–**d**) HPC parameters without perturbation at ${\bar{\sigma }}_{22}=0.00435$ GPa and (**e**–**h**) with 20% perturbation at ${\bar{\sigma }}_{22}=0.00432$ GPa. Distribution of (**e**) perturbation parameter $\lambda_{\mathrm{pert}}$, (**b**,**f**) total stress $\sigma_{22}$ in GPa, (**c**,**g**) equivalent plastic strain $\alpha^{\mathrm{HPC}}$ in HPC phase and (**d**,**h**) phase-field parameter *q* in the HPC phase, at a macroscopic strain of ${\bar{\varepsilon }}_{22}=0.175‰$.

It is observed that the resulting stress–strain curves for reinforced HPC (red and black curves) exhibit characteristics that match those of the curves for pure HPC (green and gray curves) without perturbation. However, the macroscopic stresses in the RVE and cuboid of reinforced HPC (red and black curves) are higher in comparison to the related RVE and cuboid of pure HPC (green and gray curves), see Figure 18a. Likewise, in Figure 18b, the curves obtained from the simulations of pure HPC with perturbations (cyan and yellow curves) are positioned below the curves plotted for reinforced HPC with perturbations (blue and brown curves). This phenomenon can be attributed to the additional stiffness incorporated into the reinforced HPC due to the inclusion of the fiber phase within the phenomenological material model. It is important to note that the curves obtained from simulations considering pure HPC (cyan and yellow curves) and reinforced HPC (blue and brown curves) with perturbations of HPC parameters initially have similar stress–strain characteristics, but later they begin to diverge from each other. Therefore, the curves for simulations of reinforced HPC exhibit a higher load-bearing capacity before failure, see Figure 18b. Indeed, this is the intended result, obtained by introducing the random perturbation of specifically chosen parameters, i.e., κ,μ and $\psi^{c}$. Here, the observed effect is primarily ascribed to the random perturbation of critical fracture energies within the concrete matrix. Figure 19e shows the distribution of random perturbations in the cuboid used for the simulations of macroscopic BVP I. The simulation results of macroscopic BVP I with a cuboid of reinforced HPC without perturbation, see Figure 19b–d and with 20% perturbation in the HPC parameters, see Figure 19f–h, are plotted. The influence of random perturbations is clearly seen when analyzing the macroscopic responses for macroscopic BVP I by comparing the results of both simulations using a cuboid of reinforced HPC with and without perturbations. This comparison includes the distribution of total stress $\sigma_{22}$ in the *y*-direction, the equivalent plastic strain $\alpha^{\mathrm{HPC}}$ in the HPC phase and the phase-field parameter *q* in the HPC phase within cuboids of reinforced HPC without perturbation at macroscopic stress of ${\bar{\sigma }}_{22}=0.00435$ GPa, see Figure 19b–d, and with 20% perturbation of HPC parameters at a macroscopic stress of ${\bar{\sigma }}_{22}=0.00432$ GPa, see Figure 19f–h, and at a macroscopic strain of ${\bar{\varepsilon }}_{22}=0.175‰$.

#### 4.2.3. Numerical Simulations of Macroscopic BVPs II–V

In this section, the simulation results of macroscopic BVPs, from II to V, focusing on the macroscopic stress–strain diagram are compared and discussed. The plots comparing the distribution of various quantities over the cuboid for all macroscopic BVPs, from II to V, are included in Appendix A.2.

*Macroscopic BVP II—transversely constrained uniaxial tensile test:*

**Figure 20:**
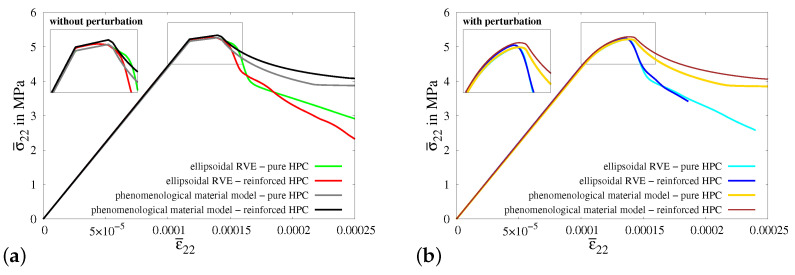
**Macroscopic BVP II**: comparison of macroscopic stress–strain characteristics with the results of virtual experiment II using ellipsoidal RVEs (**a**) without perturbation and (**b**) with 20% perturbation of HPC parameters.

**Figure A1:**
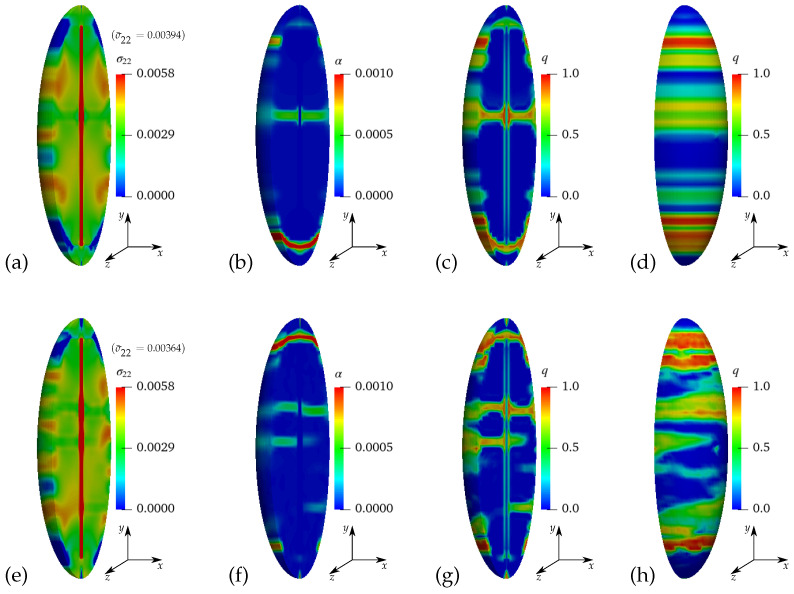
**Virtual experiment II**: uniaxial tensile test transversely constrained using ellipsoidal RVE of reinforced HPC, (**a**–**d**) HPC parameters without perturbation at ${\bar{\sigma }}_{22}=0.00394$ GPa and (**e**–**h**) with 20% perturbation at ${\bar{\sigma }}_{22}=0.00364$ GPa: distribution of (**a**,**e**) microscopic stress $\sigma_{22}$ in GPa, (**b**,**f**) microscopic equivalent plastic strain α and microscopic phase-field parameter *q* (**c**,**g**) cross-sectional view and (**d**,**h**) front view at macroscopic strain ${\bar{\varepsilon }}_{22}=0.175‰$.

Macroscopic BVP II is simulated by applying homogeneous boundary conditions on a cuboid, specifically for the uniaxial tensile test considering constrained boundaries in the transverse direction, see Figure A5a. The resulting macroscopic stress–strain characteristics for the simulations of virtual experiment II using ellipsoidal RVEs and macroscopic BVP II using cuboids of pure and reinforced HPC without perturbation, see Figure 20a, and with 20% perturbation of HPC parameters, see Figure 20b, are compared. In this instance, a plateau at the top of the stress–strain plots is observed across all simulation results, which is a consequence of the transversely constrained boundary conditions. In the resulting curves for simulations involving perturbation, the plateau appears to be smoother compared to simulations conducted without perturbation. However, stress degradation occurs more rapidly in the macroscopic response of ellipsoidal RVEs when compared to the stress–strain behavior observed in cuboids at the macroscopic level. As a result, the stress–strain plots for virtual experiment II and macroscopic BVP II exhibit noticeable deviations from each other, see Figure 20a,b. This is due to the initial damage localization in the soft fiber–concrete interface zone in the ellipsoidal RVE, compare Figure A1g and Figure A5h.

*Macroscopic BVP III—uniaxial tensile test with transverse compression:*

**Figure 21:**
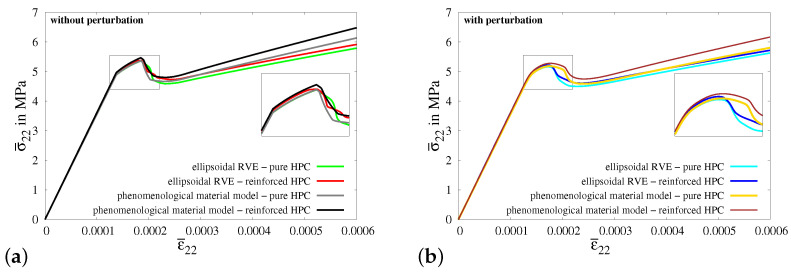
**Macroscopic BVP III**: comparison of macroscopic stress–strain characteristics with the results of virtual experiment III using ellipsoidal RVEs (**a**) without perturbation and (**b**) with 20% perturbation of HPC parameters.

**Figure A2:**
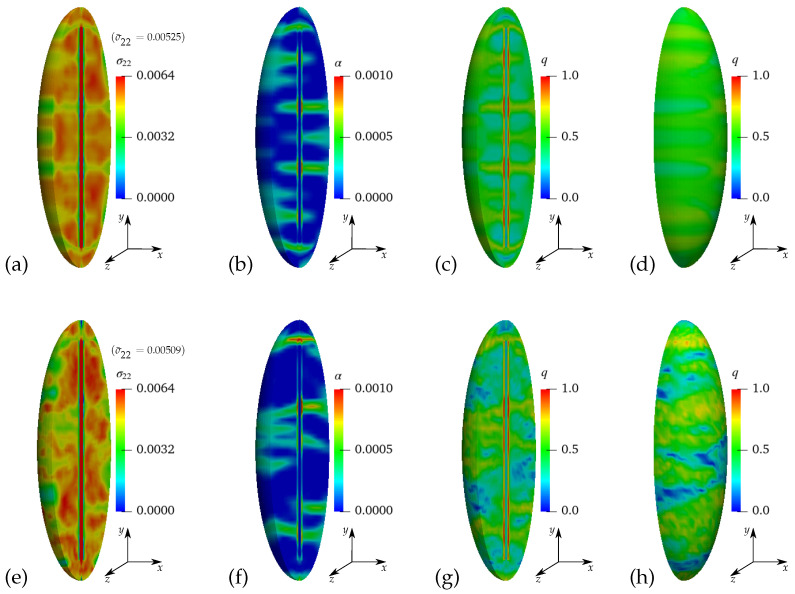
**Virtual experiment III**: uniaxial tensile test with transverse compression using ellipsoidal RVE of reinforced HPC, (**a**–**d**) HPC parameters without perturbation at ${\bar{\sigma }}_{22}=0.00525$ GPa and (**e**–**h**) with 20% perturbation at ${\bar{\sigma }}_{22}=0.00509$ GPa: distribution of (**a**,**e**) microscopic stress $\sigma_{22}$ in GPa, (**b**,**f**) microscopic equivalent plastic strain α and microscopic phase-field parameter *q* (**c**,**g**) cross-sectional view and (**d**,**h**) front view at macroscopic strain ${\bar{\varepsilon }}_{22}=0.4‰$.

In the simulations of macroscopic BVP III, homogeneous boundary conditions for a uniaxial tensile test along with compression applied on the surfaces of the cuboid in the transverse directions. This is accomplished by imposing a compressive displacement on the transverse surfaces of the cuboid, as shown in Figure A6a. The amount of displacement applied is calculated to ensure that the strains on the transverse boundaries satisfy the condition ${\bar{\varepsilon }}_{11}={\bar{\varepsilon }}_{33}=-2\;\nu \;{\bar{\varepsilon }}_{22}$. The resultant macroscopic stress–strain behaviors observed in all simulations for virtual experiment III and macroscopic BVP III, see Figure 21a,b, have a similar material response. Damage initiates once the stress reaches the tensile strength threshold. However, as the transverse compression increases, there is a subsequent rise in the stresses. This occurs because the applied transverse compression provides significant resistance to the further evolution of damage by aiding in the closure of cracks. However, as the strain increases, the stress in the cuboids of pure HPC surpass the stress in RVEs of reinforced HPC (compare gray and red curves in Figure 21a and yellow, blue and red curves in Figure 21b). This is related to the geometrical structure of the RVE, which includes a softer interface zone with a lower tensile strength, which does not exist in the cuboid. Damage tends to initiate earlier in this zone than in the other regions of the RVE, thereby reducing the overall stress in the RVE’s macroscopic response. This can be observed by comparing the distribution of the phase-field parameters in Figure A2c,g with Figure A6d,h. Similar to the observations made in the comparative analysis of experiment I, it is noticeable that the inclusion of fibers results in added stiffness in the stress–strain curves for both simulations using RVEs and cuboids of reinforced HPC, see Figure 21a,b.

*Macroscopic BVP IV—uniaxial tensile test in the transverse direction:*

**Figure 22:**
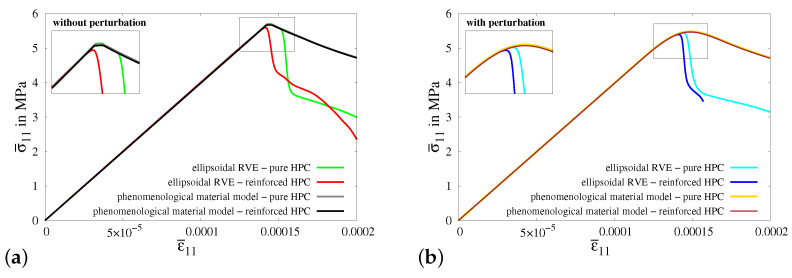
**Macroscopic BVP IV**: comparison of macroscopic stress–strain characteristics with the results of virtual experiment IV using ellipsoidal RVEs (**a**) without perturbation and (**b**) with 20% perturbation of HPC parameters.

**Figure A3:**
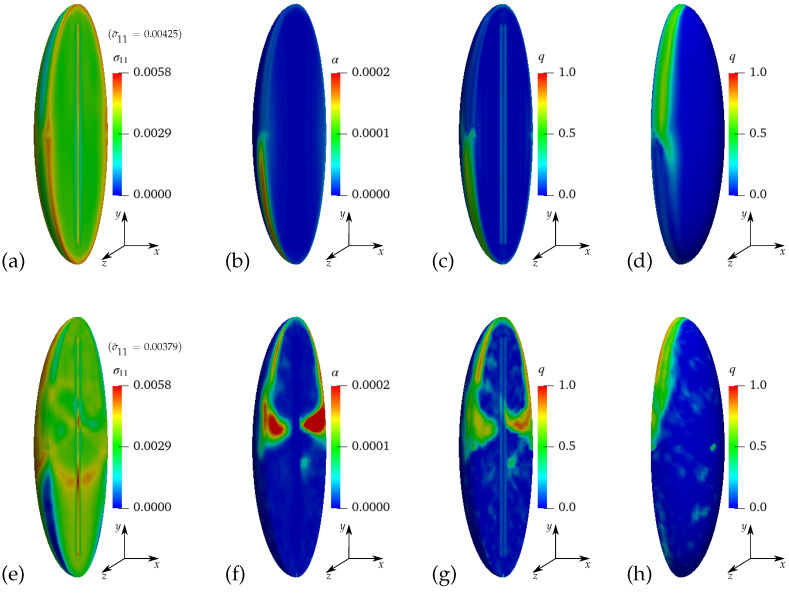
**Virtual experiment IV**: uniaxial tensile test in transverse direction using ellipsoidal RVE of reinforced HPC, (**a**–**d**) HPC parameters without perturbation at ${\bar{\sigma }}_{11}=0.00425$ GPa and (**e**–**h**) with 20% perturbation at ${\bar{\sigma }}_{11}=0.00379$ GPa: distribution of (**a**,**e**) microscopic stress $\sigma_{11}$ in GPa, (**b**,**f**) microscopic equivalent plastic strain α and microscopic phase-field parameter *q* (**c**,**g**) cross-sectional view and (**d**,**h**) front view at macroscopic strain ${\bar{\varepsilon }}_{11}=0.15‰$.

**Figure A5:**
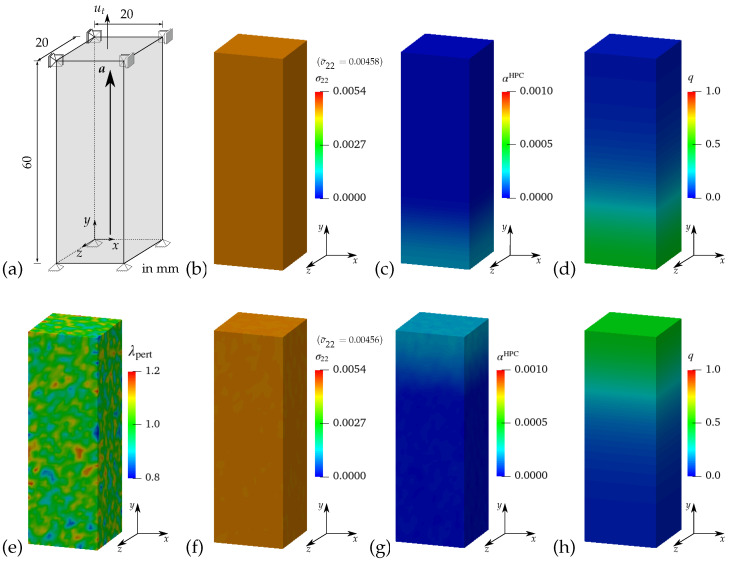
Phenomenological material model applied to (**a**) **macroscopic BVP II**, (**b**–**d**) HPC parameters without perturbation at ${\bar{\sigma }}_{22}=0.00458$ GPa and (**e**–**h**) with 20% perturbation at ${\bar{\sigma }}_{22}=0.00456$ GPa: distribution of (**e**) perturbation parameter $\lambda_{\mathrm{pert}}$, (**b**,**f**) total stress $\sigma_{22}$ in GPa, (**c**,**g**) equivalent plastic strains $\alpha^{\mathrm{HPC}}$ in HPC phase and (**d**,**h**) phase-field parameter *q* in HPC phase at macroscopic strain ${\bar{\varepsilon }}_{22}=0.175‰$.

**Figure A6:**
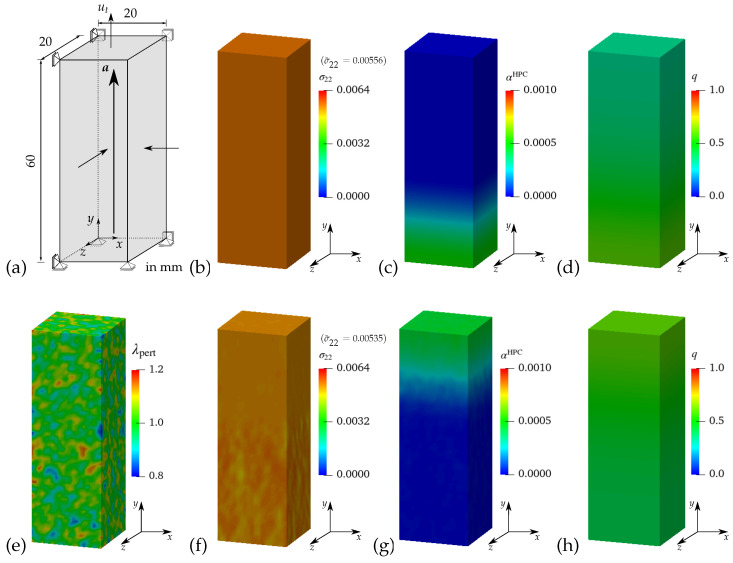
Phenomenological material model applied to (**a**) **macroscopic BVP III**, (**b**–**d**) HPC parameters without perturbation at ${\bar{\sigma }}_{22}=0.00556$ GPa and (**e**–**h**) with 20% perturbation at ${\bar{\sigma }}_{22}=0.00535$ GPa: distribution of (**e**) perturbation parameter $\lambda_{\mathrm{pert}}$, (**b**,**f**) total stress $\sigma_{22}$ in GPa, (**c**,**g**) equivalent plastic strain $\alpha^{\mathrm{HPC}}$ in HPC phase and (**d**,**h**) phase-field parameter *q* in HPC phase at macroscopic strain ${\bar{\varepsilon }}_{22}=0.4‰$.

**Figure A7:**
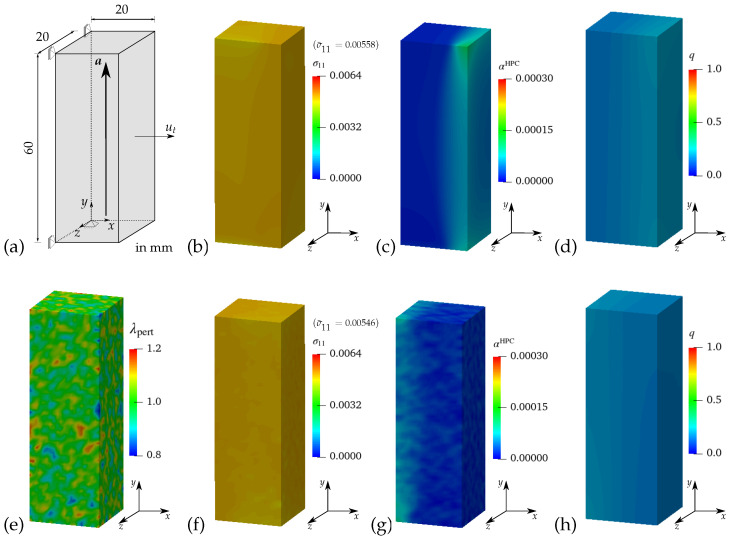
Phenomenological material model applied to (**a**) **macroscopic BVP IV**, (**b**–**d**) HPC parameters without perturbation at ${\bar{\sigma }}_{11}=0.00558$ GPa and (**e**–**h**) with 20% perturbation at ${\bar{\sigma }}_{11}=0.00546$ GPa: distribution of (**e**) perturbation parameter $\lambda_{\mathrm{pert}}$, (b, f) total stress $\sigma_{11}$ in GPa, (**c**,**g**) equivalent plastic strain $\alpha^{\mathrm{HPC}}$ in HPC phase and (**d**,**h**) phase-field parameter *q* in HPC phase at macroscopic strain ${\bar{\varepsilon }}_{11}=0.15‰$.

Figure A7a illustrates a cuboid along with the boundary conditions representative of a uniaxial tensile test in the transverse direction, i.e., perpendicular to the fiber direction, which are applied in the simulations of macroscopic BVP IV. Consequently, the embedded fibers do not significantly impact the overall material behavior of concrete in terms of providing additional stiffness and enhancing the load-bearing capacity. This characteristic is prominently observed in the stress–strain behavior for the simulations of macroscopic BVP IV with cuboids of both pure and reinforced HPC (compare gray and black curves in Figure 22a and yellow and brown curves in Figure 22b). Similar to the previous discussion, simulations of virtual experiment IV also exhibit a rapid reduction in stiffness due to the presence of a softer fiber–concrete interface zone in the reinforced RVE used for the virtual experiments. This effect becomes significant in this case because the loading direction is transverse to the fiber–concrete interface zone around the fiber (compare Figure A3g and Figure A7h). Nevertheless, there are significant differences in the macroscopic stress–strain characteristics obtained from simulations of virtual experiment IV and macroscopic BVP IV. 

*Macroscopic BVP V—shear test:*

**Figure 23:**
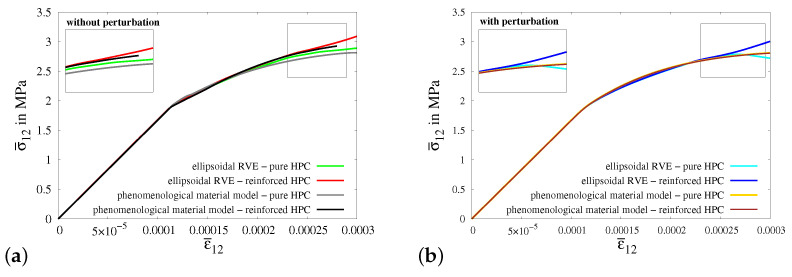
**Macroscopic BVP V**: comparison of macroscopic stress–strain characteristics with the results of virtual experiment V using ellipsoidal RVEs (**a**) without perturbation and (**b**) with 20% perturbation of HPC parameters.

**Figure A8:**
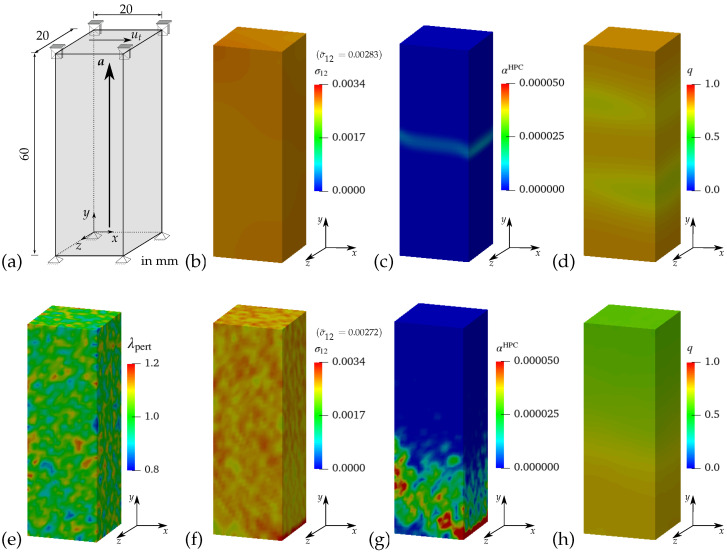
Phenomenological material model applied to (**a**) **macroscopic BVP V**, (**b**–**d**) HPC parameters without perturbation at ${\bar{\sigma }}_{12}=0.00283$ GPa and (**e**–**h**) with 20% perturbation at ${\bar{\sigma }}_{12}=0.00272$ GPa: distribution of (**e**) perturbation parameter $\lambda_{\mathrm{pert}}$, (**b**,**f**) total stress $\sigma_{12}$ in GPa, (**c**,**g**) equivalent plastic strain $\alpha^{\mathrm{HPC}}$ in HPC phase and (**d**,**h**) phase-field parameter *q* in HPC phase at macroscopic strain ${\bar{\varepsilon }}_{12}=0.25‰$.

Macroscopic BVP V is conducted to simulate a macroscopic homogeneous strain state for a shear test. The geometry and boundary conditions for macroscopic BVP V are depicted in Figure A8a. A comparative analysis of the resulting macroscopic stress–strain behavior is conducted for the simulations of virtual experiment V using ellipsoidal RVEs and macroscopic BVP V using cuboids of pure and reinforced HPC without perturbation and with 20% perturbation of HPC parameters, see Figure 23a,b.

The macroscopic stress–strain curves obtained from all four types of simulations of virtual experiment V, as well as for macroscopic BVP V, exhibit distinctive and meaningful characteristics, see Figure 23a,b. The resulting curves display a nearly identical behavior initially, before diverging at higher strain values. The macroscopic stress levels in the curves for simulations using reinforced HPC are greater than those in the curves for simulations involving pure HPC. Furthermore, the effect of perturbation can be seen clearly comparing the stress–strain results in Figure 23a,b.

## 5. Conclusions

In the initial section of the paper, an elasto-plastic phase-field model (micro-mechanical model) for simulating fractures in concrete materials is presented. The model is calibrated and validated through a comparison of experimental data with numerical results obtained from simulations of pullout tests considering different lengths of a single steel fiber embedded within HPC. These findings not only validate the accuracy of the calibrated material parameters but also serve as a foundational basis for simulations of virtual experiments using the homogenization approach. The calibrated parameters, the constructed ellipsoidal RVEs and the incorporation of concrete phase heterogeneity collectively result in a realistic representation of steel-fiber-reinforced HPC along the preferred fiber direction. Simulations of various types of virtual experiments allow for an understanding of complex interactions and failure behavior within the individual phases of heterogeneous materials at the microscopic level.

Furthermore, a phenomenological material model is presented. Therein, separate energy functions that individually characterize the behavior of the embedded steel fibers and HPC matrix are used. The use of an additive form of the energy functions makes it possible to simulate the macroscopic behavior of both pure and reinforced HPC by adjusting the values of the phase fraction. The effective utilization of different yield criteria for the fibers and HPC matrix enhances the capability of the presented model in capturing the comprehensive material behavior of fiber-reinforced HPC. These criteria account for the unique nonlinearities that exist within each phase. The concept of numerically integrating the fibers and calibrating the phenomenological (macroscopic) model using the macroscopic response obtained from virtual experiments based on RVEs circumvents the complexity associated with employing direct homogenization techniques in conjunction with the phase-field approach. Moreover, implementing different combinations of fiber orientations and distributions can also be seamlessly achieved using this approach.

The observed additional macroscopic stress level in the simulation results of BVPs using cuboids of fiber-reinforced HPC validates the efficiency of the model in capturing the material behavior of fiber-reinforced HPC. Moreover, it is observed during the comparisons that the macroscopic stress–strain characteristics are significantly influenced by the introduced heterogeneity through random perturbations in HPC parameters. It is notable that the macroscopic stress–strain characteristics in virtual experiments II and IV and macroscopic BVPs do not align precisely with each other. This discrepancy arises from the difference in the construction of RVEs, which involve three materials (steel fiber, interface zone, HPC matrix), as opposed to a cuboid where the fibers are represented numerically. To improve these results, it could be beneficial to incorporate all types of macroscopic BVPs during the calibration and regularization of the phenomenological material model.

The concept of representing fibers phenomenologically through a one-dimensional elasto-plasticity model enables the numerical definition of embedded fibers. This not only simplifies the complexity and time required for geometrically representing fibers but also conserves computational efforts. Moreover, it also opens up the possibility of implementing an orientation distribution function to accommodate the orientations and distribution of all the embedded fibers with little effort. Therefore, this numerical approach holds potential for further development to comprehensively capture the overall material behavior of fiber-reinforced HPC. This can be accomplished by utilizing the calibrated numerical parameters and incorporating realistic distributions and orientations of fibers used in the experiments, as planned for future work.

## Figures and Tables

**Table 1:** Mechanical properties of steel and HPC materials and calibrated values of material parameters used for the simulation of the fiber pullout test, cf. [66,68,81].

	*E*	ν	$f_{t}$	$f_{c}$	$\psi_{t}^{c}$	$\psi_{c}^{c}$	$y_{0}$	$\beta_{p}$	*h*	*l*	*m*	ζ
	**GPa**	**−**	**MPa**	**MPa**	**MPa**	**MPa**	**−**	**−**	**mm**	**−**	**−**	
Steel	210	0.3	1150	–	0.4	0.4	660	0	130	0.4	0.6	0.5
Interface	39.976	0.192	–	–	2 × 10^−4^	2 × 10^−4^	6.263	0	0	0.4	0.3	0.5
HPC	39.976	0.192	5.7	112	4.2 × 10^−4^	0.12	6.263	0.5218	2000	0.4	0.6	0.5

**Table 2:** The material parameters used in the phenomenological material model for simulations of macroscopic BVPs, cf. [66,68,81,89].

$E^{\mathrm{HPC}}$	$\nu^{\mathrm{HPC}}$	$\psi_{t}^{c,\mathrm{HPC}}$	$\psi_{c}^{c,\mathrm{HPC}}$	$y_{0}^{\mathrm{HPC}}$	$\beta_{p}$	$h^{\mathrm{HPC}}$	*m*	ζ	$E^{F}$	$v^{F}$	$y_{0}^{F}$	$h^{F}$
**GPa**	**−**	**MPa**	**MPa**	**MPa**	**−**	**mm**	**−**	**−**	**GPa**	**−**	**MPa**	**MPa**
39.976	0.192	4.2 × 10^−4^	0.12	6.263	0.5218	2000	0.6	0.5	210	0.003	660	130

**Figure A4:**
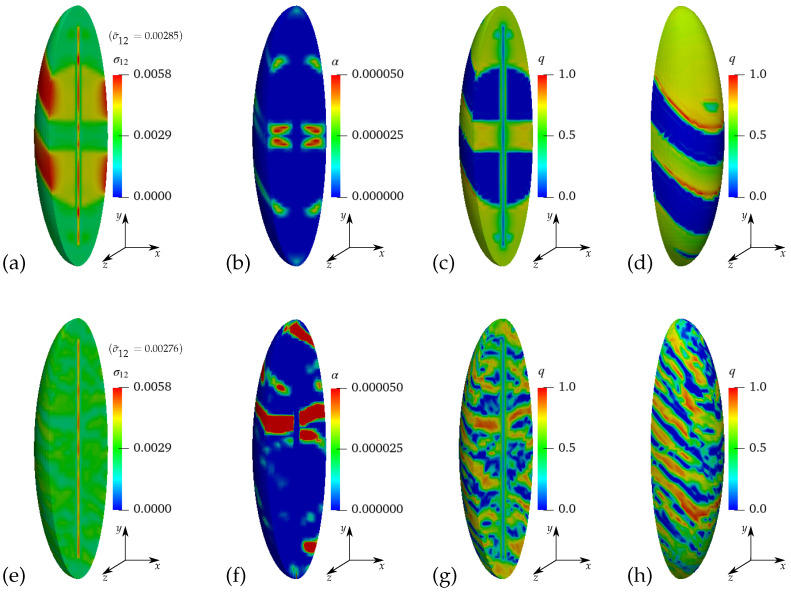
**Virtual experiment V**: shear test using an ellipsoidal RVE of reinforced HPC, (**a**–**d**) HPC parameters without perturbation at ${\bar{\sigma }}_{12}=0.00285$ GPa and (**e**–**h**) with 20% perturbation at ${\bar{\sigma }}_{12}=0.00276$ GPa: distribution of (**a**,**e**) microscopic stress $\sigma_{12}$ in GPa, (**b**,**f**) microscopic equivalent plastic strain α and microscopic phase-field parameter *q* (c, g) cross-sectional view and (**d**,**h**) front view at macroscopic strain ${\bar{\varepsilon }}_{12}=0.25‰$.

## Data Availability

The experimental data are given in [66].

## Appendix A

### Appendix A.1. Simulation Results of Virtual Experiments II–V

Here, the distribution comparing various quantities obtained from the simulations conducted in virtual experiments II to V are analyzed. For additional information on the boundary value problems associated with each virtual experiment, please refer to Section 3.3.

*Virtual experiment II—uniaxial tensile test transversely constrained:*

The simulation results of virtual experiment II using a reinforced ellipsoidal RVE without perturbation of HPC parameters at macroscopic stress ${\bar{\sigma }}_{22}=0.00394$ GPa, see Figure A1a–d and with 20% perturbation at macroscopic stress ${\bar{\sigma }}_{22}=0.00364$ GPa, see Figure A1e–h, of material parameters are plotted at macroscopic strain ${\bar{\varepsilon }}_{22}=0.175‰$. Therein, the distribution of microscopic stress $\sigma_{22}$ in *y*-direction in GPa, see Figure A1a,e, microscopic equivalent plastic strain α, see Figure A1b,f and microscopic phase-field parameter *q* in cross-sectional view, see Figure A1c,g and in front view, see Figure A1d,h are shown. In the distribution of phase-field parameter *q*, compare Figure A1d,h, the effect of random perturbations of material parameter can be easily observed.

*Virtual experiment III—uniaxial tensile test with transverse compression:*

The simulation results of virtual experiment III using a reinforced ellipsoidal RVE without perturbation of HPC parameters at macroscopic stress ${\bar{\sigma }}_{22}=0.00525$ GPa, see Figure A2a–d and with 20% perturbation at macroscopic stress ${\bar{\sigma }}_{22}=0.00509$ GPa, see Figure A2e–h, are plotted at macroscopic strain ${\bar{\varepsilon }}_{22}=0.4‰$. Therein, the distribution of microscopic stress $\sigma_{22}$ in *y*-direction in GPa, see Figure A2a,e, microscopic equivalent plastic strain α, see Figure A2b,f and microscopic phase-field parameter *q* in cross-sectional view, see Figure A2c,g and in front view, see Figure A2d,h are shown. In the distribution of phase-field parameter *q*, e.g., compare Figure A2d,h, the effect of random perturbations of material parameter can be easily observed. In addition to that, the evolution of phase-field parameter *q*, Figure A2c,d,g,h, is lower in magnitude as compare to the stress in *y*-direction in GPa, see Figure A2a,e which indicates the delay in failure of concrete material due to transverse compression.

*Virtual experiment IV—uniaxial tensile test in transverse direction:*

The simulation results of virtual experiment IV using a reinforced ellipsoidal RVE without perturbation of HPC parameters at macroscopic stress ${\bar{\sigma }}_{11}=0.00425$ GPa, see Figure A3a–d and with 20% perturbation at macroscopic stress ${\bar{\sigma }}_{11}=0.00379$ GPa, see Figure A3e–h, of material parameters are plotted at macroscopic strain ${\bar{\varepsilon }}_{11}=0.15‰$. Therein, the distribution of microscopic stress $\sigma_{22}$ in *x*-direction in GPa, see Figure A3a,e, microscopic equivalent plastic strain α, see Figure A3b,f and microscopic phase-field parameter *q* in cross-sectional view, see Figure A3c,g and in front view, see Figure A3d,h, are shown.

*Virtual experiment V—shear test:*

The simulation results of virtual experiment IV using a reinforced ellipsoidal RVE without perturbation of HPC parameters at macroscopic stress ${\bar{\sigma }}_{12}=0.00285$ GPa, see Figure A4a–d and with 20% perturbation at macroscopic stress ${\bar{\sigma }}_{12}=0.00276$ GPa, see Figure A4e–h, of material parameters are plotted at macroscopic strain ${\bar{\varepsilon }}_{12}=0.25‰$. Therein, the distribution of microscopic stress $\sigma_{12}$ in GPa, see Figure A4a,e, microscopic equivalent plastic strain α, see Figure A4b,f and microscopic phase-field parameter *q* in cross-sectional view, see Figure A4c,g and in front view, see Figure A4d,h are shown. The effect of perturbation of material parameters can be easily observed in the distribution of phase-field parameter *q*, compare Figure A4c,d and Figure A1g,h, respectively.

### Appendix A.2. Simulations Results of Macroscopic BVPs II–V

Here, the distribution comparing various quantities obtained from the simulations conducted in macroscopic BVPs II to V are analyzed. For additional information on the boundary value problems associated with each virtual experiment, please refer to Section 4.2.

*Macroscopic BVP II—uniaxial tensile test transversely constrained:*

The macroscopic responses obtained from the simulations of macroscopic BVP II using cuboids of reinforced HPC, are plotted in terms of the distribution of total stress $\sigma_{22}$ in *y*-direction, the equivalent plastic strain $\alpha^{\mathrm{HPC}}$ in HPC phase and the phase-field parameter *q* in HPC phase at macroscopic strain ${\bar{\varepsilon }}_{22}=0.175‰$. This is depicted for cuboids of reinforced HPC without perturbations at macroscopic stress ${\bar{\sigma }}_{22}=0.00458$ GPa in Figure A5b–d, and with a 20% perturbation of HPC parameters at macroscopic stress ${\bar{\sigma }}_{22}=0.00456$ GPa in Figure A5f–h. Therein, the minor effect of random perturbations is particularly observed when comparing the distribution of the equivalent plastic strain $\alpha^{\mathrm{HPC}}$ in Figure A5c,g, as well as the phase-field parameter *q* in Figure A5d,h.

*Macroscopic BVP III—uniaxial tensile test with transverse compression:*

The simulation results of of macroscopic BVP III using cuboids without perturbation of HPC parameters at macroscopic stress ${\bar{\sigma }}_{22}=0.00556$ GPa, see Figure A6a–d and with 20% perturbation at macroscopic stress ${\bar{\sigma }}_{22}=0.00535$ GPa, see Figure A6e–h, of material parameters are plotted at macroscopic strain ${\bar{\varepsilon }}_{22}=0.4‰$. The macroscopic responses are plotted in terms of the distribution of total stress $\sigma_{22}$ in *y*-direction, the equivalent plastic strain $\alpha^{\mathrm{HPC}}$ in HPC phase and the phase-field parameter *q* in HPC phase at macroscopic strain ${\bar{\varepsilon }}_{22}=0.4‰$. As discussed earlier, the applied transverse compression resists the the evolution of damage. This clarifies the less damage apparent in the distribution of the phase-field parameter *q* in HPC phase, see Figure A6d,h. The influence of perturbations in the material parameters of HPC phase is evident in the macroscopic stress-strain curves plotted in Figure 21b, as well as in the comparisons involving the distribution of total stress $\sigma_{22}$, see Figure A6b,f and the equivalent plastic strain $\alpha^{\mathrm{HPC}}$ in HPC phase, see Figure A6c,g.

*Macroscopic BVP IV—uniaxial tensile test in transverse direction:*

The macroscopic responses obtained from the simulations of macroscopic BVP IV using cuboids of reinforced HPC, are plotted in terms of the distribution of total stress $\sigma_{11}$ in *x*-direction, the equivalent plastic strain $\alpha^{\mathrm{HPC}}$ in HPC phase and the phase-field parameter *q* in HPC phase at macroscopic strain ${\bar{\varepsilon }}_{11}=0.15‰$. The results for simulations with cuboids of reinforced HPC without perturbations at macroscopic stress ${\bar{\sigma }}_{11}=0.00558$ GPa in Figure A7b–d, and with a 20% perturbation of HPC parameters at macroscopic stress ${\bar{\sigma }}_{22}=0.00546$ GPa in Figure A7f–h. A similar effect of random parameter perturbation is observed in the macroscopic response of cuboids for macroscopic BVP IV, consistent with previous cases. This effect is reflected in the scattered distribution of total stress $\sigma_{11}$, see Figure A7b,f, as well as a mirrored pattern in the distribution of equivalent plastic strain $\alpha^{\mathrm{HPC}}$ and the phase-field parameter *q* in the HPC phase, compare Figure A7c with Figure A7g, and Figure A7d with Figure A7h, respectively.

*Macroscopic BVP V—shear test:*

The influence of parameter perturbation is clearly noticeable during the comparison of the macroscopic responses of macroscopic BVP V using cuboid of reinforced HPC without perturbation at macroscopic stress ${\bar{\sigma }}_{12}=0.00283$ GPa, see Figure A8b–d and with 20% perturbation of HPC parameters at macroscopic stress ${\bar{\sigma }}_{12}=0.00272$ GPa, see Figure A8f–h. The comparison includes distribution of total stress $\sigma_{12}$, the equivalent plastic strain $\alpha^{\mathrm{HPC}}$ in HPC phase and the phase-field parameter *q* in HPC phase obtained from the simulation of macroscopic BVP V using cuboids of reinforced HPC with and without perturbation of HPC parameters. In this context, a notably random and scattered distribution of total stress $\sigma_{12}$, see Figure A8b,f and the equivalent plastic strain $\alpha^{\mathrm{HPC}}$ within the HPC phase, Figure A8c,g is evident. It is noticeable that the evolution in the phase-field parameter *q* in HPC phase occurs in the different areas of cuboid, see Figure A8d,h.
